# Multidimensional analyses identify genes of high priority for pancreatic cancer research

**DOI:** 10.1172/jci.insight.174264

**Published:** 2025-01-07

**Authors:** Zeribe C. Nwosu, Heather M. Giza, Maya Nassif, Verodia Charlestin, Rosa E. Menjivar, Daeho Kim, Samantha B. Kemp, Peter Sajjakulnukit, Anthony Andren, Li Zhang, William K.M. Lai, Ian Loveless, Nina Steele, Jiantao Hu, Biao Hu, Shaomeng Wang, Marina Pasca di Magliano, Costas A. Lyssiotis

**Affiliations:** 1 Department of Molecular & Integrative Physiology, University of Michigan, Ann Arbor, Michigan, USA.; 2 Department of Chemistry and Biochemistry, University of Notre Dame, Notre Dame, Indiana, USA.; 3 Cellular and Molecular Biology Program and; 4 Department of Surgery, University of Michigan, Ann Arbor, Michigan, USA.; 5 Department of Molecular Biology and Genetics, Cornell University, Ithaca, New York, USA.; 6 Center for Bioinformatics, Department of Public Health Sciences, Henry Ford Health, Detroit, Michigan, USA.; 7 Department of Computational Mathematics, Science, and Engineering; Medical Imaging and Data Integration Lab; Michigan State University, East Lansing, Michigan, USA.; 8 Henry Ford Pancreatic Cancer Center, Department of Surgery, Detroit, Michigan, USA.; 9 Department of Pathology and Oncology, Wayne State University, Detroit, Michigan, USA.; 10 Department of Pharmacology and Toxicology, Michigan State University, East Lansing, Michigan, USA.; 11 Department of Internal Medicine, Medical School,; 12 Department of Pharmacology, Medical School,; 13 Department of Medicinal Chemistry, College of Pharmacy,; 14 Rogel Cancer Center,; 15 Department of Cell and Developmental Biology, and; 16 Department of Internal Medicine, Division of Gastroenterology, University of Michigan, Ann Arbor, Michigan, USA.

**Keywords:** Gastroenterology, Oncology, Cancer, Glucose metabolism, Molecular genetics

## Abstract

Pancreatic ductal adenocarcinoma (PDAC) is a drug-resistant and lethal cancer. Identification of the genes that consistently show altered expression across patient cohorts can expose effective therapeutic targets and strategies. To identify such genes, we separately analyzed 5 human PDAC microarray datasets. We defined genes as “consistent” if upregulated or downregulated in 4 or more datasets (adjusted *P* < 0.05). The genes were subsequently queried in additional datasets, including single-cell RNA-sequencing data, and we analyzed their pathway enrichment, tissue specificity, essentiality for cell viability, and association with cancer features, e.g., tumor subtype, proliferation, metastasis, and poor survival outcome. We identified 2,010 consistently upregulated and 1,928 downregulated genes, of which more than 50% to our knowledge were uncharacterized in PDAC. These genes spanned multiple processes, including cell cycle, immunity, transport, metabolism, signaling, and transcriptional/epigenetic regulation — cell cycle and glycolysis being the most altered. Several upregulated genes correlated with cancer features, and their suppression impaired PDAC cell viability in prior CRISPR/Cas9 and RNA interference screens. Furthermore, the upregulated genes predicted sensitivity to bromodomain and extraterminal (epigenetic) protein inhibition, which, in combination with gemcitabine, disrupted amino acid metabolism and in vivo tumor growth. Our results highlight genes for further studies in the quest for PDAC mechanisms, therapeutic targets, and biomarkers.

## Abstract 2

This study provides multi-dataset analyses of differential genes in pancreatic cancer and reveals high priority consistent genes associated with tumor metastasis, proliferation, prognosis and therapy

## Graphical abstract

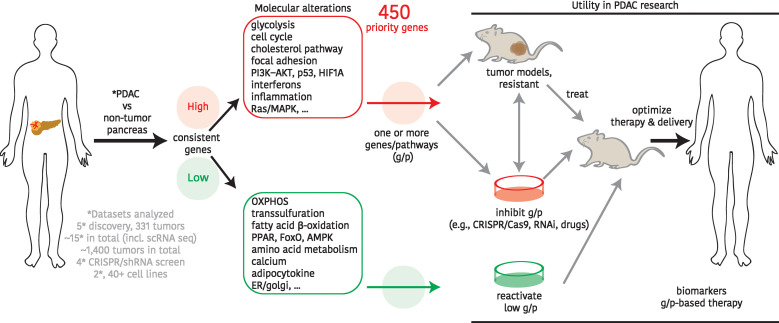

## Introduction

Pancreatic ductal adenocarcinoma (PDAC) is an aggressive, highly metastatic and drug-resistant disease, and is one of the most lethal tumors (1). Prior studies have converged on *KRAS*, *TP53*, *SMAD4*, and *CDKN2A* as the most frequently mutated genes in PDAC (2–5). Oncogenic mutations, notably in the *KRAS* gene, dictate drug sensitivity and drive tumor initiation and progression (6, 7). Studies of gene expression profiles have defined 2 to 4 major subtypes of PDAC (2–4, 8–10) and these subtypes impact therapeutic response and patient overall survival. Although numerous studies have generated datasets on gene expression from PDAC, genes that are consistently expressed at high or low levels across the multiple datasets are still largely unknown. Hitherto, mainly due to limited samples, most studies validating the relevance of genes have relied on data from a small cohort of patients and often use as few as one dataset. This approach underestimates the scope of PDAC gene changes, especially as several of those validated genes may not be reproducibly changed in independent datasets and therefore may not be the key drivers of PDAC. To facilitate future PDAC research, we herein sought to identify genes with the highest probability of being relevant in basic and translational contexts. We first analyzed 5 published PDAC microarrays to determine genes with similar expression pattern across most of the datasets. We further checked the expression of those genes in over 20 different datasets, including microarrays (of PDAC and other gastrointestinal cancers — namely colon, gastric, and liver cancers); bulk tumor and single-cell RNA sequencing (scRNA-seq) data; and normal tissues and cancer cell line gene expression data of drug response and CRISPR/Cas9 and shRNA screens. Overall, we identified several genes that are strongly dysregulated in PDAC, and we show that several of these genes are expressed mainly in PDAC in comparison with other gastrointestinal cancers. Using cell lines and mouse models, we demonstrate the utility of the consistent genes in predicting drug response. Our work identifies core differential genes in PDAC and will facilitate gene prioritization for basic, translational, and clinical research, thus paving the way for new discoveries that could improve PDAC therapy.

## Results

### The consistently differential genes in PDAC.

**Figure 1:**
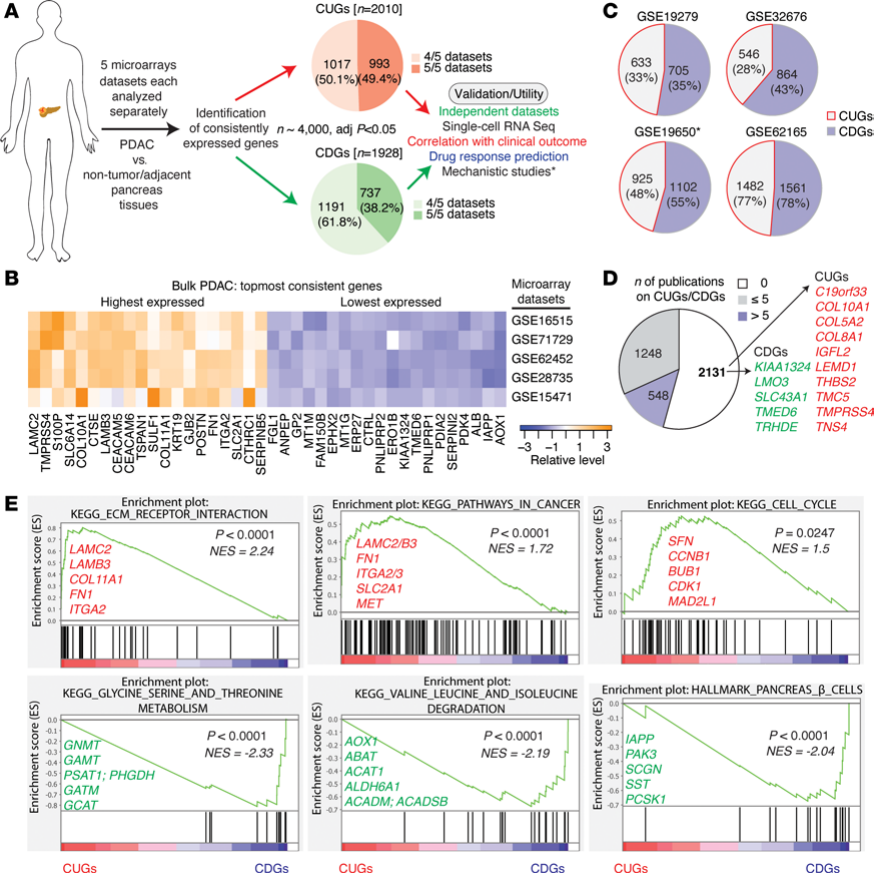
Consistently upregulated or downregulated genes in human pancreatic tumors. (**A**) Schematic overview illustrating the identification and potential utility of the consistent genes. Five PDAC microarray datasets were used for the identification of the genes and “consistent” was defined as genes upregulated or downregulated in at least 4 datasets (adjusted *P* < 0.05). CUGs, consistently upregulated genes; CDGs, consistently downregulated genes. See Methods (*Identification of the consistent genes*) or Supplemental Figure 1B for the sample size of each dataset. *Not the focus of this study. (**B**) Topmost 20 highly and lowly expressed genes in PDAC (i.e., CUGs and CDGs, respectively) based on the sum of expression rank in the 5 datasets. (**C**) Number of CUGs and CDGs that showed the same high or low expression pattern, respectively, in the independent microarray datasets GSE19279, GSE32676, GSE19650, and GSE62165. *Dataset of premalignant tumor stages. See Supplemental Methods for sample sizes/types of the independent datasets. (**D**) Pie chart indicating the distribution of consistent genes (i.e., CUGs and CDGs) in PDAC relative to the number (*n*) of prior publications on the genes (0, ≤5, and >5) as observed via PubMed search. Highlighted in red are topmost CUGs with no prior publication; in green are topmost CDGs with no prior publication. (**E**) Gene set enrichment analysis (GSEA) plots of Kyoto Encyclopedia of Genes and Genomes (KEGG) pathways or Hallmark associated with the consistent genes. The plots were generated using only the 3,938 genes that are consistent (adjusted *P* < 0.05 in tumor versus nontumor comparison in at least 4 of 5 datasets). The gene list used for GSEA was ranked by the sum of the expression score across the 5 datasets. NES, normalized enrichment score.

To identify the most consistent differential genes in PDAC, and genes potentially important in basic or translational contexts, we analyzed over 20 publicly accessible datasets, including microarrays (of tumors, normal tissues, cell lines), RNA-seq, as well as CRISPR/Cas9 and RNA interference (RNAi) data (Figure 1A and Supplemental Figure 1A; supplemental material available online with this article; https://doi.org/10.1172/jci.insight.174264DS1).

We started with 5 PDAC tumor microarray datasets that have sample sizes ranging from 36 to 145 (Supplemental Figure 1B). Each dataset was analyzed separately and only genes with adjusted *P* values of less than 0.05 were selected as statistically significant per dataset. We selected and defined a gene as “consistent” if it was upregulated in at least 4 of the 5 datasets or downregulated across the same number of datasets. Thus, we eliminated the likelihood of selecting a gene by chance. Based on our selection criteria, we identified 2,010 consistently upregulated genes (hereafter CUGs) and 1,928 consistently downregulated genes (CDGs). As many as 993 (49% of CUGs) and 737 (38% of CDGs) were consistent in all 5 datasets (Figure 1A), indicating that these genes have a very reproducible expression pattern in PDAC regardless of the various sources of the datasets or the underlying tumor samples. Notably, we ranked these genes using the product of their log_2_(fold change) and –log(*P* value) per dataset and used the sum of the ranked scores across the 5 datasets to determine the genes with the highest or lowest expression. We found that the most highly expressed CUGs included *LAMC2*, *TMPRSS4*, *S100P*, *SLC6A14*, *COL10A1*, *CTSE*, *LAMB3*, *CEACAM5/6*, and glucose transporter *SLC2A1* (Figure 1B), and these appeared within the top 1%–5% of highly expressed genes in all 5 datasets (Supplemental Table 1A). In contrast, the CDGs with the lowest expression included *AOX1*, *IAPP*, albumin (*ALB*), *SERPINI2*, *PDK4*, and *PNLIPRP1/2* (Figure 1B), and these also showed the lowest expression across the 5 datasets (Supplemental Table 1A).

We analyzed 4 additional PDAC patient cohorts and found that in these cohorts, 28% to approximately 80% of the already defined CUGs and CDGs were also high and low, respectively (Figure 1C). Furthermore, the topmost genes in the initial 5 datasets also appeared topmost in at least 3 of the 4 independent datasets (Supplemental Figure 1C), supporting the notion that at least the topmost CUGs and CDGs can be expected to be differentially expressed in any PDAC cohort. In the initial 5 datasets, we also identified 2,722 additional genes (*n* = 1,063 up and 1,659 down) that showed consistent expression in 3 of the 5 datasets (adjusted *P* < 0.05) (Supplemental Table 1B and Supplemental Figure 1D). Several of these genes, although not emphasized in our analysis, are also either similarly high or low in the 4 independent cohorts. They included well-known candidates in cancer such as *ALDOC*, *HIF1A* (in glycolysis), *SNAI2*, *ZEB1* (in epithelial-mesenchymal transition), monocarboxylate transport 4 (*SLC16A3*), *MMP2*, *GPNMB*, *KRT17*, and *INHBA* (all of which were upregulated), as well as downregulated genes such as *GATA4*, *ADH1A*, *PTF1A*, *CBS*, and genes encoding pancreatic digestive enzymes (e.g., *PRSS1*, *CPA1*, and *CPB1*). As expected, many CUGs and CDGs have been mechanistically studied or proposed as PDAC biomarkers or therapeutic targets. However, we found no substantial prior PDAC publication on over 50% of the approximately 4,000 consistent genes (Figure 1D and Supplemental Table 2), indicating potentially untapped opportunities for new discoveries.

To determine whether the consistent genes reflect molecular changes typically seen in cancer, we performed pathway enrichment and gene ontology (GO) analyses. Indeed, known oncogenic changes such as “cell cycle,” “extracellular matrix receptor interaction,” “pathways in cancer,” “focal adhesion,” “p53,” and “transcription factor binding” emerged as upregulated. These were accompanied by evidence of disrupted pancreatic β cell homeostasis and profound metabolic changes (Figure 1E and Supplemental Figure 1E). Interactome and disease ontology analyses further associated the consistent genes with precancerous conditions, PDAC, and other malignancies (Supplemental Figure 2, A and B). Collectively, these data reveal the extensive nature of molecular changes in pancreatic tumors.

### Consistent genes are present in multiple pathways underlying PDAC.

**Figure 2:**
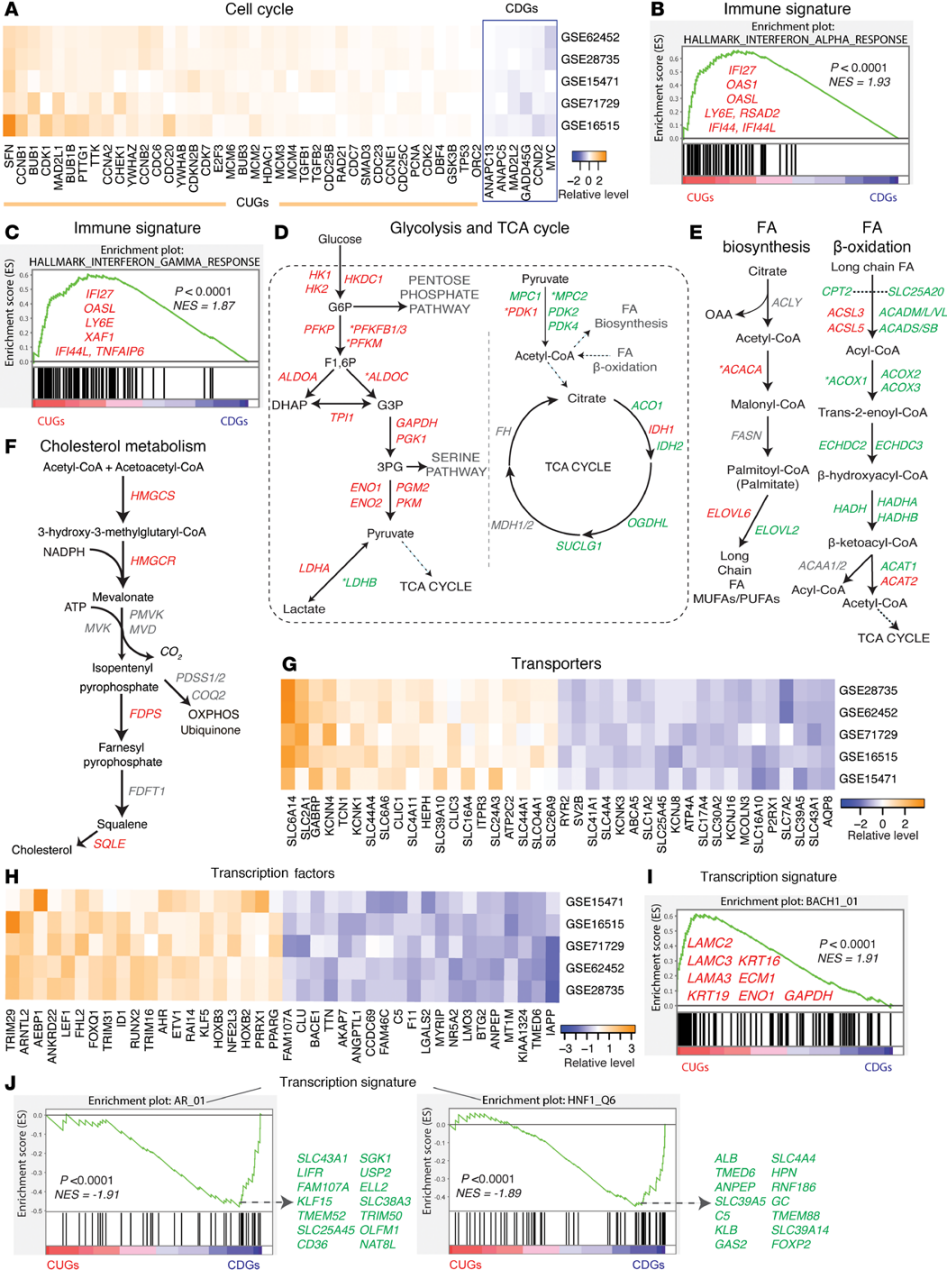
Consistent genes are components of multiple pathways. (**A**) Heatmap showing cell cycle components with consistent expression pattern. (**B** and **C**) Gene set enrichment analysis (GSEA) plots indicating upregulated (**B**) IFN-α and (**C**) IFN-γ signatures. The listed genes are the most highly upregulated in the pathways shown. (**D**–**F**) Schematic depictions of consistent genes in the (**D**) glycolytic pathway and the tricarboxylic acid (TCA) cycle, (**E**) fatty acid (FA) biosynthesis and breakdown (β-oxidation), and (**F**) cholesterol metabolism. (**G**) Heatmap showing the most consistently expressed nutrient and ion transporters (top 20 up-/downregulated). (**H**) Heatmap showing the most consistently expressed transcription factors (top 20 up-/downregulated). (**I** and **J**) GSEA plots of transcriptional signature, indicating (**I**) upregulated BACH1_01 signature and (**J**) downregulated AR_01 and HNF_Q6 signatures. The genes in red are among the topmost of the enriched BACH1_01 signature, whereas genes in green are among the topmost downregulated in the AR_01 and HNF_Q6 plots, respectively. In **D** and **E**, *indicates genes that were consistent in 3 of 5 datasets; all other genes displayed are consistent in 4 or more datasets (in red – CUGs, in green – CDGs). Genes in gray were not captured as consistent and are shown based on their known position/role in the respective pathways. NES, normalized enrichment score.

We wanted to determine the specific genes that make up the pathway enrichment plots (Figure 1E and Supplemental Figure 1E). This is because, although it is important to identify individual differentially expressed genes, the appearance of multiple genes in a pathway is a more robust indicator of the broader involvement of that pathway in PDAC. We found that several consistent genes featured across molecular pathways/profiles, e.g., cell cycle, metabolism/transport, signaling, immunity, epigenetics, and transcription factor genes, ranging from 45 genes in cell cycle to 723 transcription factors (Supplemental Table 3). Most of the identified cell cycle genes (39 of 45) were upregulated (CUGs) — the topmost being *SFN*, *CCNB1*, *BUB1*, *CDK1*, and *MAD2L1*. Only 6 CDGs appeared under cell cycle (Figure 2A) and those were *ANAPC13*, *ANAPC5*, *MAD2L2*, *GADD45G*, *CCND2*, and surprisingly *MYC* — a gene often implicated as a cancer driver. In immune pathways, most of the identified genes (60 of 76) were CUGs, notably *CXCL5*, *ITGA3*, *CCL20*, *IFI44L*, and *IFI27*. We also identified IFN-α and IFN-γ, TNF-α/NF-κB, “inflammatory response,” and IL-2/STAT5 signaling as strongly enriched immune hallmarks in PDAC (Figure 2, B and C, and Supplemental Figure 3A).

In metabolism, glycolysis genes emerged as the most consistently high in PDAC, but most components of the tricarboxylic acid (TCA) cycle, which links glycolysis to oxidative phosphorylation (OXPHOS), were downregulated or inconsistently expressed (Figure 2D). Other notably upregulated metabolic genes included *SULF1/2* (in glycan metabolism), *GPX2/8* (in glutathione metabolism), and *NQO1* and *NOX4* (in oxidation/reduction reactions) (Supplemental Table 3). In contrast, the de novo serine biosynthesis pathway, which branches off from glycolysis, is downregulated in PDAC (Supplemental Figure 3B). Furthermore, the OXPHOS “hallmark” (Supplemental Figure 3A) and components of fatty acid β-oxidation (Figure 2E) were downregulated, altogether pointing to an altered mitochondrial metabolism. PDAC showed the upregulation of genes in fatty acid biosynthesis and cholesterol metabolism, although key genes like *ACLY*, *FASN*, *MVK*, and *FDFT1* did not meet our criteria for consistency (Figure 2, E and F). Several genes in the transsulfuration pathway and amino acid metabolism were downregulated, with the notable exception of tryptophan metabolism, where *IDO1*, *TDO2*, *KMO*, and *KYNU* were upregulated (Supplemental Figure 3, C and D).

Transporters enable cells to acquire or shuttle nutrients for metabolism and growth and are potential targets in cancer (11). We found that the topmost upregulated transporters were *SLC6A14* (a neurotransmitter/amino acid transporter), *SLC2A1* (a glucose transporter), *GABRP*, *KCNN4*, and *TCN1* (a cobalamin transporter), whereas *P2RX1*, *SLC7A2*, *SLC39A5*, *SLC43A1*, and *AQP8* showed the lowest expression (Figure 2G). For most of the transporters, we found limited to no studies on their expression and function in PDAC. In signal transduction, HIF-1, p53, MAPK, Hippo, and AGE-RAGE signaling were among the upregulated pathways (Supplemental Figure 3E), whereas cGMP-PKG, PPAR, and AMPK signaling mostly had downregulated genes (Supplemental Figure 3F).

With respect to transcription factors, the highly expressed components included *TRIM29*, *ARNTL2*, *AEBP1* *ANKRD22*, and *LEF1* (in Wnt signaling), whereas those with the lowest expression were *IAPP*, *KIAA1324*, *MT1M*, and *ANPEP* (Figure 2H). Furthermore, the transcription signature for BACH1 (Figure 2I) also emerged as highly upregulated, whereas AR and HNF1 signatures were downregulated (Figure 2J). Epigenetic targets such as *KDM5B*, *EZH2*, *HDAC1*, *HELLS*, *DNMT1*, *KDM2A/1B*, and histone protein–encoding genes *HIST1H2BC*, *HIST1H2BD*, *HIST1H2BE*, *HIST1H4H*, and *HIST2H2BE* also emerged as upregulated in PDAC, whereas *SIRT3*, *CXXC1*, *ING3*, *KDM8*, *CITED2/4*, and *KAT2B* (Supplemental Figure 3G) were among the CDGs with the lowest expression. These data highlight specific genes across the multiple pathways that are altered in PDAC.

### Consistent genes show tumor-specific expression.

We next wanted to determine whether the consistent genes are tumor-specific or merely reflect the tumor microenvironment cell gene profile. Previous studies have approached the question of specificity of gene expression in PDAC by using microdissection to distinguish signatures of tumors from that of stroma (10, 12). We used datasets from those prior studies, but applied a different approach. Namely, our approach first determined consistent genes from 4–5 datasets before assessing the expression of those genes in normal pancreatic tissues relative to other normal tissues from 3 datasets, i.e., Human Protein Atlas, Genotype-Tissue Expression (GTEx) Project, and NCBI Gene Expression Omnibus (GEO) GSE71729 (Supplemental Figure 4, A–C). With this approach, we expected tumor-specific CUGs to be low in a normal pancreas relative to other tissues and tumor-specific CDGs to show the opposite pattern. These criteria were fulfilled by 1,333 (66%) CUGs and 1,384 (72%) CDGs in at least 2 datasets (Supplemental Table 4A). Glycolysis genes *HK1*, *GAPDH*, *ENO1*, *LDHA*, and *PKM*, and cell cycle genes (except *CCND2*) were among the most tumor-specific genes. Others included *TSC22D1*, *B2M*, *S100A6*, and *CFL1*, as well as *TNS4*, *LEMD1*, *CORO2A*, *ASAP2*, *MTMR11*, and *FXYD5*, which to our knowledge are still poorly understood in PDAC. For the CDGs, among the most highly expressed in normal pancreas were *CEL*, *GP2*, *REG1A*, *REG1B*, *REG3G*, *PRSS3*, *PNLIPRP1*, and *PLA2G1B* (Supplemental Table 4A). Of note, these CDGs were highly expressed in the “exocrine-like” tumor subtype defined by Collison et al. (3), which may suggest that “exocrine-like” PDACs are well differentiated.

**Figure 3:**
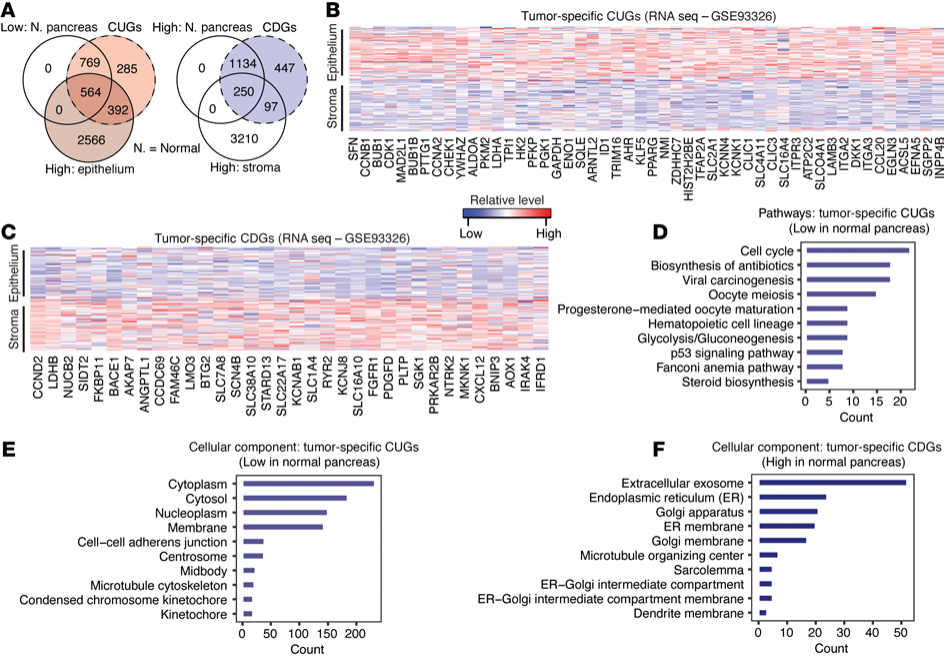
Consistent genes show a tumor-specific or non–tumor-specific expression pattern. (**A**) Venn diagram showing overlap between CUGs and genes with low expression in normal pancreas and/or highly expressed in epithelium compared with stroma from the laser microdissection dataset (GSE93326). Right: Venn diagram showing overlap between CDGs and genes highly expressed in normal pancreas and/or highly expressed in the stroma (low in epithelium). Gene expression in the normal pancreas was determined by comparing pancreatic tissue gene expression to that of other normal tissues in the Human Protein Atlas (HPA, *n* = 42 other tissue types), Genotype-Tissue Expression (GTEx, *n* = 33 other tissue types), and GSE71729 (Moffitt) datasets (normal tissues: pancreas, *n* = 46; liver, *n* = 27; lung, *n* = 19; lymph node, *n* = 10; spleen, *n* = 11). Genes that showed a similar expression pattern in at least 2 of HPA, GTEx, or GSE71729 were selected. (**B** and **C**) Heatmaps showing (**B**) topmost tumor-specific CUGs differentially expressed in the tumor epithelium samples, and (**C**) tumor-specific CDGs that show low expression in the tumor epithelium samples of the laser capture microdissection dataset GSE93326 (*n* = 65 pairs of tumor epithelium and stroma samples). (**D**) Pathway annotation of the 564 tumor-specific CUGs (high expression in the epithelium and low expression in normal pancreas). (**E** and **F**) Gene ontology for cellular component of the (**E**) 564 core CUGs and (**F**) the 250 core CDGs.

Differential analysis of laser capture microdissection RNA-seq dataset GSE93326 (12) revealed that several CUGs and CDGs were significantly expressed (adjusted *P* < 0.01) in PDAC epithelium versus stroma (Supplemental Table 4A). Analysis of the overlap between consistent genes that were expressed in normal pancreas and genes from epithelium versus stroma comparison revealed 564 CUGs that were low in the stroma and normal pancreas and 250 CDGs that were high in the stroma and normal pancreas (Figure 3A). We considered these to be “core tumor-specific” genes in PDAC (Figure 3, A–C). Another 392 CUGs (~20%) were high in the epithelium and, although not low in normal pancreas in our analysis, several of these, including *KRT7*, *AREG*, *S100A2*, *LYZ*, *TFF1*, *AGR2*, and *CTSE*, were described as tumor-specific by Moffitt et al. (10). Approximately 38% of CUGs (*n* = 769) were only low in normal pancreas and some of those genes (e.g., *ACTB*, *MYL6/10*, *NDRG1*, *TIMP1*, *SLC39A10*, *TGFB2*, *NOX4*, *MMP9*, *ADAM17*, *PEA15*, *SEPT7*, and *PGM2*) were more highly expressed in the stroma relative to epithelium. A variety of known genes emerged among CDGs that were high in normal pancreas (e.g., *ASNS*, *MT1E*, *MT1G*, *MT1F*, *GPT2*, *GNMT*, *GAMT*, *PDK4*, *PSAT1*, *SLC25A25*, and *SLC43A1*), high in the stroma (e.g., *CDO1*, *CSF1*, *EPAS1*, *GHR*, *KLF9*, *LYVE1*, *NCOA1*, *OGN*, and *SELENOP*), or did not overlap with genes high in pancreas or stroma (e.g., *ACADM*, *ACADSB*, *ANKRD7*, *ANKRD11*, *FXYD1*, *FXYD7*, *KANK1*, and *KANK3*) (Supplemental Table 4A).

Pathway annotation analysis of the “core tumor-specific” CUGs identified “cell cycle/division,” “glycolysis,” “p53,” and “progesterone-mediated oocyte maturation” (consisting of *CCNB1*, *CDK1*, *HSP90AA1*, *MAD2L1*, *CCNB2*, *BUB1*, *CCNA2*, *CDK*, and *CDC25B* genes) among pathways that are low in normal pancreas but elevated in PDAC (Figure 3D). In contrast, based on the core CDGs, “protein processing in endoplasmic reticulum” (ER) (e.g., *UBE2D4*, *ATF4*, *HERPUD1*, *MAN1A2*, and *UBE2J1*), “FoxO signaling pathway” (e.g., *IGF1R*, *SGK1*, *GABARAPL1*, *CCND2*, and *NLK*), and “transcriptional misregulation in cancer” (e.g., *IGF1R*, *NUPR1*, *CCND2*, *PDGFA*, and *RXRA*) are low in PDAC relative to normal pancreas (Supplemental Table 4A). Gene ontology analysis showed that the tumor-specific CUGs are associated with cytoplasmic, membrane, and nuclear/nucleoplasmic activities (Figure 3E), whereas tumor-specific CDGs are associated with ER, Golgi apparatus, and exosomes (Figure 3F). These data suggest core changes that, when present in the normal pancreas, may give rise to or permit the progression of PDAC. Of note, several CUGs and CDGs were also strongly expressed in PDAC compared with other gastrointestinal tumors, namely, colon, gastric, and liver cancer (Supplemental Figure 4D and Supplemental Table 4, B and C), further supporting PDAC-specific expression patterns in some cases.

**Figure 4:**
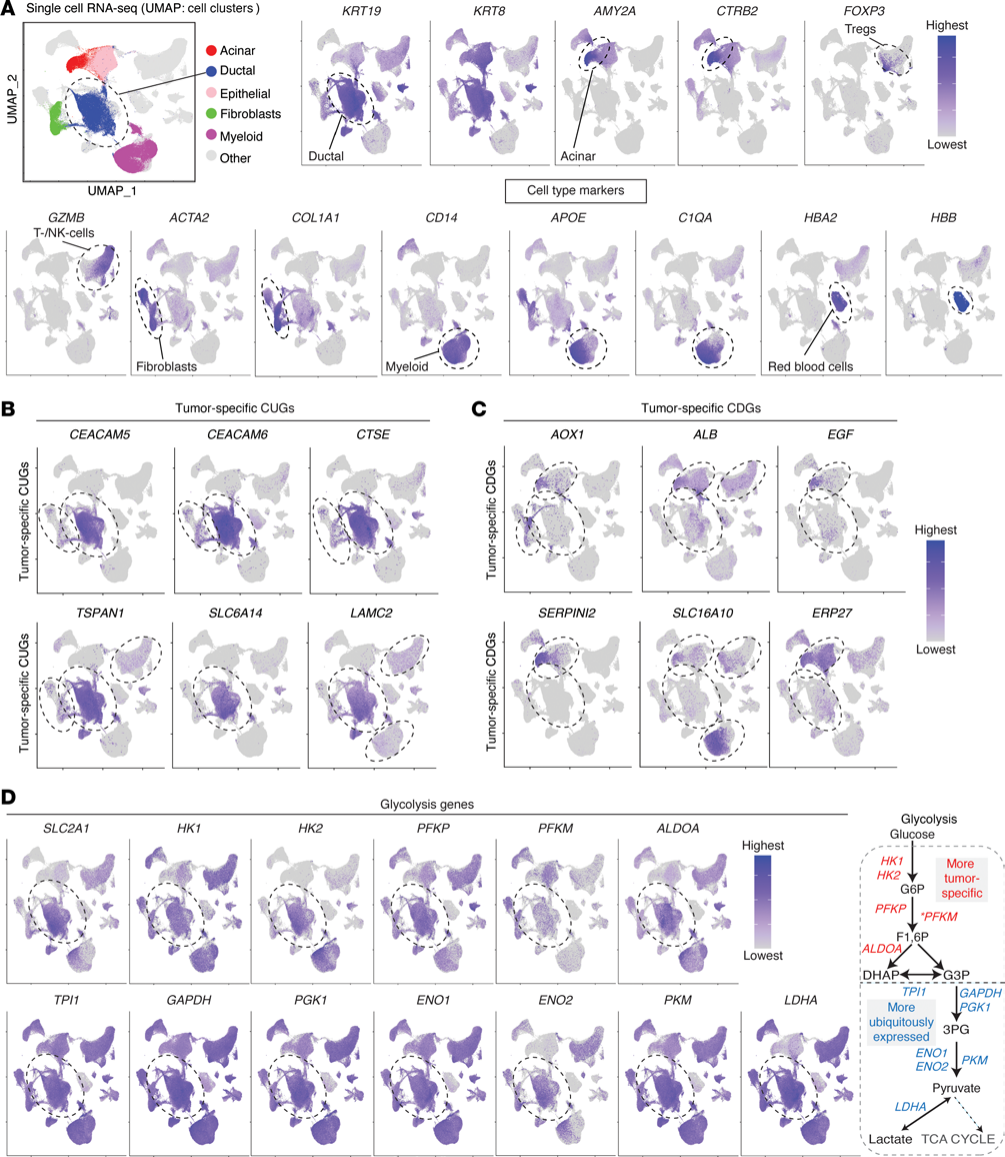
scRNA-seq data showing the expression of the consistent genes in tumor and surrounding cell population. (**A**) Uniform manifold approximation and projections (UMAPs) of single-cell RNA-sequencing (scRNA-seq) data of human PDAC, depicting various cell populations and cell type marker plots. *KRT19*, keratin 19; *KRT8*, keratin 8 (pancreatic ductal epithelial markers); *AMY2A*, amylase α2A; *CTRB2*, chymotrypsinogen B2 (pancreatic acinar cell markers); *FOXP3*, forkhead box P3 (regulatory T cell [Treg] marker); *GZMB*, granzyme B (cytotoxic T/natural killer [NK] cell marker); *ACTA2*, actin α2 smooth muscle; *COL1A1*, collagen type I α1 chain (fibroblast markers); *CD14*, cluster of differentiation 14; *APOE*, apolipoprotein E; *C1QA*; complement C1q A chain (macrophage/myeloid cell markers); *HBA2*, hemoglobin subunit α2; *HBB*, hemoglobin subunit β (red blood cell markers). Sample size: *n* = 61 primary tumors. (**B**) UMAPs showing cell populations expressing CUGs that showed tumor-specific upregulation in microarrays and laser capture microdissection dataset (in Figure 3) and (**C**) CDGs that showed tumor-specific downregulation. (**D**) UMAPs showing consistently upregulated glycolysis genes that emerged as more tumor-specific (upper row, except *PFKM*) or ubiquitously expressed in most other cell types (lower row). On the right is a schematic summary showing the glycolysis steps associated with the displayed genes. *Consistent in 3 of 5 datasets shown in Figure 1.

To further gain insight into the expression of the consistent genes relative to tumor microenvironment cell composition, we harnessed our published scRNA-seq profile of human PDAC (13–17). We used various cell markers to identify the cell population to enhance specificity (Figure 4A and Supplemental Figure 5A). As expected, we validated the specificity of several consistent genes, notably CUGs such as *CEACAM5*, *CEACAM6*, *CTSE*, *TSPAN1*, and *KRT19* (Figure 4B), and CDGs like *AOX1*, *EGF*, *SLC16A10*, *SERPINI2*, and *IAPP* (Figure 4C and Supplemental Figure 5B). Considering that glycolysis is well known to be upregulated in most cancers, we looked at the expression of the pathway genes at scRNA resolution. Intriguingly, glycolysis genes showed variable and, in several cases, ubiquitous expression (Figure 4D). Such ubiquitous expression was shown by IFN-α–inducible protein 27 (*IFI27*) (Supplemental Figure 5C), despite its emergence among the top-ranked CUGs in PDAC and as tumor-specific in other comparisons. These data collectively support the notion that a remarkable proportion of the identified consistent genes show PDAC tissue–specific expression, whereas some may also be expressed in other cell types.

### Consistent genes correlate with cancer features.

**Figure 5:**
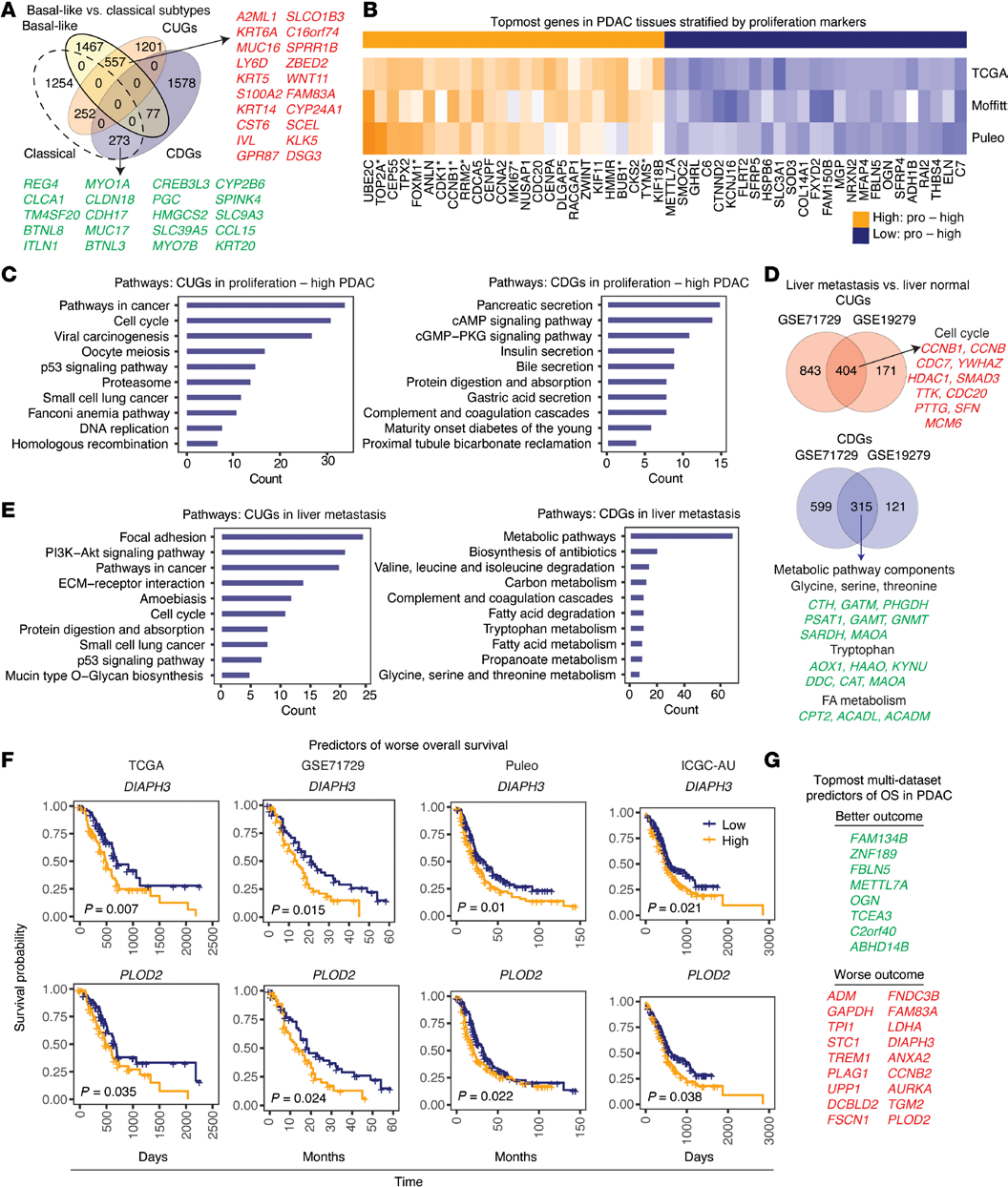
Consistent genes correlate with poor cancer prognostic features. (**A**) Venn diagram showing overlap between CUGs or CDGs and genes expressed by PDAC basal-like or classical subtypes. The basal-like versus classical subtype genes included are statistically upregulated or downregulated (*P* < 0.05) in 2 or more datasets, namely, TCGA (*n* = 31 basal-like vs. 31 classical), GSE71729 (Moffitt dataset, *n* = 27 basal-like vs. 27 classical), and Puleo (*n* = 64 basal-like vs. 64 classical tumor samples). Genes in red/green are selected examples. (**B**) Heatmap depicting topmost upregulated or downregulated genes in proliferation-high PDAC. *Indicates proliferation markers used for the tumor stratification and included as positive controls. Genes included are statistically up-/downregulated in proliferation-high relative to proliferation-low tumors in 2 or more of TCGA (*n* = 64 proliferation-high vs. 86 proliferation-low), GSE71729 (*n* = 77 proliferation-high vs. 68 proliferation-low), and Puleo (*n* = 99 proliferation-high vs. 210 proliferation-low) tumors (*P* < 0.05). Pro, proliferation. (**C**) Pathway annotation of 783 CUGs and 588 CDGs that overlapped with genes differentially expressed in proliferation-high vs. -low tumors in at least 2 datasets. cAMP, cyclic adenosine monophosphate; cGMP, cyclic guanosine monophosphate; PKG, protein kinase G. (**D**) Venn diagrams showing CUGs and, below, CDGs overlapping with genes in liver metastasis compared with normal liver tissues from GSE71729 and GSE19279 datasets (*P* < 0.05). See Supplemental Methods for sample sizes/types. (**E**) Pathway annotation of the CUGs and CDGs that overlapped between liver metastasis compared with normal liver tissues. PI3K, phosphoinositide 3-kinase; ECM, extracellular matrix. (**F**) Kaplan-Meier (KM) overall survival (OS) plots (log-rank test, *P* < 0.05) of genes that predicted survival in the clinical cohorts analyzed. Tumor sample size: TCGA (*n* = 146), GSE71729 (*n* = 125), Puleo (*n* = 288); and ICGC (*n* = 267). (**G**) Topmost genes that predicted OS in at least 3 of the 4 pancreatic adenocarcinoma datasets, i.e., TCGA, Puleo et al., GSE71729/Moffitt, and International Cancer Genome Consortium (IGCG) datasets. High expression of the genes in green predicts “better” outcome, whereas those in red predict “worse” outcome.

We queried the expression of the CUGs and CDGs in previously published classical and basal-like PDAC subtypes — the latter being associated with worse survival outcome (10). In addition, we correlated the genes with proliferation, metastasis, mutations, tumor grade, and patient survival outcome (Supplemental Figure 6A). To derive classical versus basal-like groups in 3 cohorts (i.e., The Cancer Genome Atlas [TCGA], Moffitt, and Puleo datasets), we stratified each dataset using 50 tumor subtype signatures published by Moffitt et al. (10), of which approximately 70% are on our list of consistent genes (Supplemental Figure 6B). We found that 28% of the CUGs (*n* = 557) were basal-like signatures, whereas 13% (*n* = 252) were classical signatures. CDGs also overlapped with signatures of both subtypes (Figure 5A and Supplemental Table 5). However, the comparison also revealed 1,503 CUGs and CDGs that were not differentially expressed (*P* > 0.05) in any of the 3 cohorts (Supplemental Figure 6C), revealing consistent genes that did not uniquely distinguish the subtypes. Examples include *TMPRSS4*, *SLC6A14*, *IFI27*, *CST1*, *STYK1* (CUGs) (Supplemental Figure 6D), and *TMED6*, *PNLIPRP1*, *SERPINI2*, *ALB*, *AOX1* (CDGs).

We also stratified the patient cohorts into proliferation-high and -low subsets (Figure 5B and Supplemental Table 5) and found 783 CUGs, including *PTTG1*, *KPNA2*, *MICALCL*, *PTGES*, *GAD1*, *KRT16*, and *CREG2*, that are upregulated in proliferation-high tumors. These proliferation-high CUGs were largely enriched in “pathways in cancer,” “cell cycle/division,” “p53,” and “proteasome” (Figure 5C). We also surprisingly observed that some CDGs, e.g., *PSAT1* in serine metabolism, *C9orf40*, *GPR3*, *PRPS2*, *SAMD1*, *IPO4*, *GIT1*, and *DFFA* were high in proliferation-high tumors despite being downregulated in PDAC, which may suggest context-dependent roles. Other than these, the majority of CDGs (*n* = 588, ~31%) were low in proliferation-high PDAC and these include superoxide dismutase 3 (*SOD3*), *OGN*, *ALDH1B*, *SLC3A1*, and complement components (*C6* and *C7*). The CDGs with low expression in proliferation-high tumors were strikingly associated with physiologic pancreatic activities, e.g., “gastric and insulin secretion” and “protein digestion/absorption” (Figure 5C). Thus, several CUGs showed direct correlation with proliferation, whereas CDGs were inversely correlated.

Metastasis accounts for over 90% of cancer deaths and is a major feature of PDAC (18). We analyzed 3 PDAC microarray datasets containing metastasized samples (mainly liver metastasis) and performed over 10 comparisons of metastatic versus primary PDAC or distant normal versus lymph node, peritoneal lung, or liver metastasis tumors (Figure 5, D and E, Supplemental Figure 7, A and B, and Supplemental Table 6A). In the metastasized tissues, CUGs such as *LAMB3*, *LGALS4*, *CEACAM6*, and *TFF2* are upregulated and pathway annotation analysis linked metastasis-correlated CUGs to “focal adhesion,” “extracellular matrix–receptor interaction,” “p53,” “cell cycle,” “proliferation,” and “mitochondrial matrix.” In contrast, CDGs that correlated with liver metastasis showed a downregulation of metabolic pathways (Figure 5, D and E). Consistent with altered expression in metastasis, we confirmed that several CUGs (e.g., *LAMC2*, *TMPRSS4*, *S100P*, *CTSE*, *LAMB3*, *CEACAM5*, *CEACAM6*, and *TSPAN1*) as well as CDGs (e.g., *SNX3*, *ANKRD11*, *CUTA*, *WDR83OS*, *NDUFB1*, *PAIP2*, *DAP*, and *UBE2B*) were respectively high or low in the epithelial cells of tumors that metastasized to the liver in the scRNA-seq data (Supplemental Table 6B).

Tumor grade is also an index of tumor progression and TCGA and the Puleo cohorts included grade annotations. Using these 2 cohorts, we identified CUGs and CDGs that distinguished Grade III (poorly differentiated) from Grade II (moderately differentiated) tumors or Grade II from Grade I (more well-differentiated) tumors (Supplemental Table 7). The accompanying pathway changes also resembled the profiles seen with metastasis or proliferation-high versus -low tumors (Supplemental Figure 7C). These data, coupled with the CUG or CDG expression profile in pancreatic intraductal papillary mucinous adenoma at the early tumor stage (19), support the notion that the consistent genes correlate with PDAC progression phases (Supplemental Figure 7D and Supplemental Table 8).

Although the most frequently mutated genes in PDAC are known (i.e., *KRAS*, *TP53*, *SMAD4*, and *CDKN2A*), the differential genes accompanying those mutations are largely undefined. Using TCGA data, we found that *KRAS* mutation, mainly G12D, had the most impact on CUG and CDG expression, followed by mutation in *CDKN2A* and *SMAD4* (adjusted *P* < 0.05; Supplemental Figure 8, A and B). *TP53* mutations had the least impact, probably due to the extensive heterogeneity (e.g., almost every couple of samples had a different *TP53* mutation). Many CUGs and CDGs were differentially expressed in tumors with KRAS mutation (G12D, G12R, or G12V) compared with those without, suggesting that mutant KRAS likely controls the expression of several consistent genes (Supplemental Figure 8C and Supplemental Table 9, A and B). The upregulated CUGs in *KRAS* G12D–mutated tumors were associated with “cell cycle,” “p53,” and “glycolysis,” whereas the downregulated CDGs were associated with other metabolic pathways, notably “fatty acid metabolism” (Supplemental Figure 8D and Supplemental Table 9C). We further used a published dataset of inducible oncogenic *Kras* signatures (7) to determine which consistent genes that correlated with KRAS in patients tumors were likely controlled by oncogenic *Kras* induction in vitro and in mice. Focusing only on genes that are differentially altered by KRAS in vivo or both in vivo and in vitro, we found that 201 CUGs from KRAS-mutated tumors in TCGA were upregulated upon *Kras* induction. These included known Ras pathway genes (*AREG*, *EREG*, and *DUSP6*), *HK1*, and *UPP1* recently shown to mediate metabolic adaptation in nutrient-restricted conditions (20). In contrast, 87 of the CDG KRAS-mutated patients’ samples were also downregulated upon KRAS induction in mice, and among these, 23 (including *PDK2*, *PDK4*, and *ACADL*) were also downregulated in vitro (Supplemental Table 9B).

To determine additional associations with clinical outcome, we performed Kaplan-Meier (KM) overall survival (OS) and univariate Cox regression analyses, respectively, using TCGA, Moffitt, Puleo, and International Cancer Genome Consortium – Australia (ICGC-AU) PDAC patient cohorts (Supplemental Table 10A, Figure 5, F and G, and Supplemental Figure 8, E–H). Over 1,600 consistent genes predicted OS in at least 1 cohort. Of these, 2 CUGs (*PLOD2* and *DIAPH3*) and CDGs (*OGN* and *C2orf40*) predicted worse outcome when high (Figure 5F) or low (Supplemental Figure 8G), respectively, in all 4 cohorts. Furthermore, several genes statistically predicted OS (*P* < 0.05) in 3 or more datasets and these include *ADM*, *UPP1*, *ZNF189*, *DCBLD2*, *PLAG1*, and glycolysis genes *ALDOA*, *GAPDH*, *PKM2*, and *LDHA* (Figure 5G and Supplemental Table 10A). Analysis of the pathways associated with CUGs that predicted poor outcome identified “PI3K-Akt,” “focal adhesion,” “proteoglycans in cancer,” “carbon metabolism,” and “glycolysis” (Supplemental Figure 8H). The consistent genes also overlapped with genes in tumors from PDAC patients that had a “bad” prognosis (Supplemental Figure 8I and Supplemental Table 10B). Collectively, these data strongly link the consistent genes with malignant tumor features, suggesting their potential relevance in pancreatic carcinogenesis.

### The consistent genes of high priority as therapeutic targets.

To derive high-priority potential therapeutic targets among the consistent genes, we focused on CUGs. We sought to determine CUGs that appear to be essential for cancer cell survival via gene interference screens (CRISPR/Cas9 and shRNA) and/or were altered at a statistically significant level (at least at *P* < 0.05) across comparisons we performed earlier (i.e., tumor-specific expression, proliferation, and survival).

**Figure 6:**
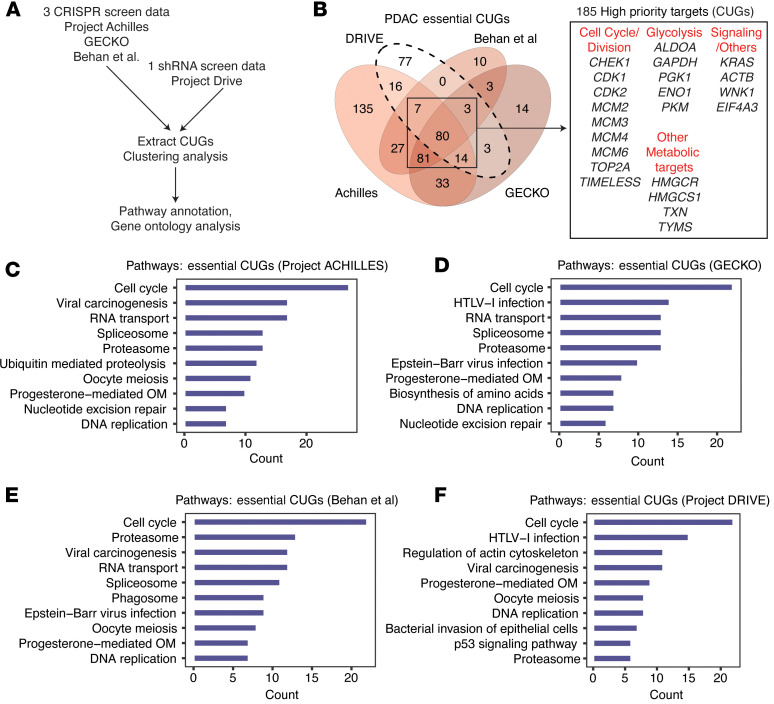
High-priority consistent genes. (**A**) Workflow for the analysis of the CRISPR/Cas9 and shRNA (collectively gene interference) screen data to identify CUGs that are potentially essential for PDAC cell survival or growth. (**B**) Venn diagram showing the overlapping essential genes in PDAC cell lines as derived from the Project Drive, Project Achilles, Behan et al., and GECKO screen data. In bold are the number of CUGs, in total 185, that overlapped as essential for survival in at least 3 of the 4 gene interference screen data. Right: Selected genes among the 185 high-priority targets. (**C**–**F**) Pathway annotation of genes that emerged as essential for PDAC survival/growth in (**C**) Project Achilles (*n* = 394 CUGs), (**D**) GECKO (*n* = 231 CUGs), (**E**) Behan (*n* = 211 CUGs), and (**F**) Project Drive (*n* = 200 CUGs). OM, oocyte maturation; HTLV, human T lymphotropic virus.

For the gene interference part, we analyzed the CRISPR/Cas9 knockout screen data from Project Achilles (21), GECKO, Behan et al. (22, 23) studies, and the shRNA knockdown data from Project Drive (24) (Figure 6A). The original corresponding publications of these data assessed the essentiality of genes for survival/growth in multiple human cancer cells and types and their results included several CUGs identified in our study (Supplemental Table 11A). In addition, the possibility of querying gene interference results from 8 to approximately 24 PDAC cell lines across 4 independent studies offered unprecedented strength to our analyses. Clustering analysis of Achilles and Project Drive (each containing 24 PDAC cell lines), the GECKO dataset (*n* = 8 PDAC cell lines), and the Behan et al. dataset (*n* = 20 PDAC cell lines) identified over 200 essential CUGs per dataset (Supplemental Table 11A). We identified 185 CUGs (herein called “high-priority therapeutic targets”) that were essential in at least 3 datasets (Figure 6B). These targets notably included glycolysis genes (*ALDOA*, *GAPDH*, and *PKM*), cell cycle/division genes (*BUB1*, *CDK1*, *CDK2*, and *MCMs*), thioredoxin (*TXN*), *KRAS*, and *ACTB*. Pathway annotation analysis revealed “cell cycle/division,” “proteasome,” “spliceosome,” and “progesterone-mediated oocyte maturation” as the most consistent changes associated with the essential genes per dataset. Others included “p53,” “regulation of actin skeleton,” and “biosynthesis of amino acids,” which contained mainly glycolysis genes (Figure 6, C–F, and Supplemental Table 11B).

Recent evidence from mechanistic studies on uridine phosphorylase 1 (*UPP1*), a CUG that emerged from our analysis, revealed that some CUGs may not appear to be essential in RNAi screens under optimal cell culture conditions. For example, the CRISPR/Cas9 knockout of *UPP1* impaired PDAC cell growth under glucose restriction, but not in glucose-replete media (20), and UPP1 did not appear to be essential in PDAC in any of the 4 gene interference screen data mentioned earlier. Thus, to capture the full spectrum of potential priority targets, we sought CUGs that are low in normal pancreas (Supplemental Table 4A), correlated with high proliferation, and correlated with either basal-like subtype (Supplemental Table 5) or predicted OS (in at least 1 dataset Supplemental Table 10A). This condition was met by 336 CUGs, of which 71 genes are part of the targets from the gene interference screen datasets (Supplemental Table 12). Thus, combined with the priority targets from the gene interference screen, we identified 450 CUGs (Supplemental Table 13) that represent high-priority targets for future studies in therapeutic or biomarker contexts.

### High-priority genes predict therapy.

**Figure 7:**
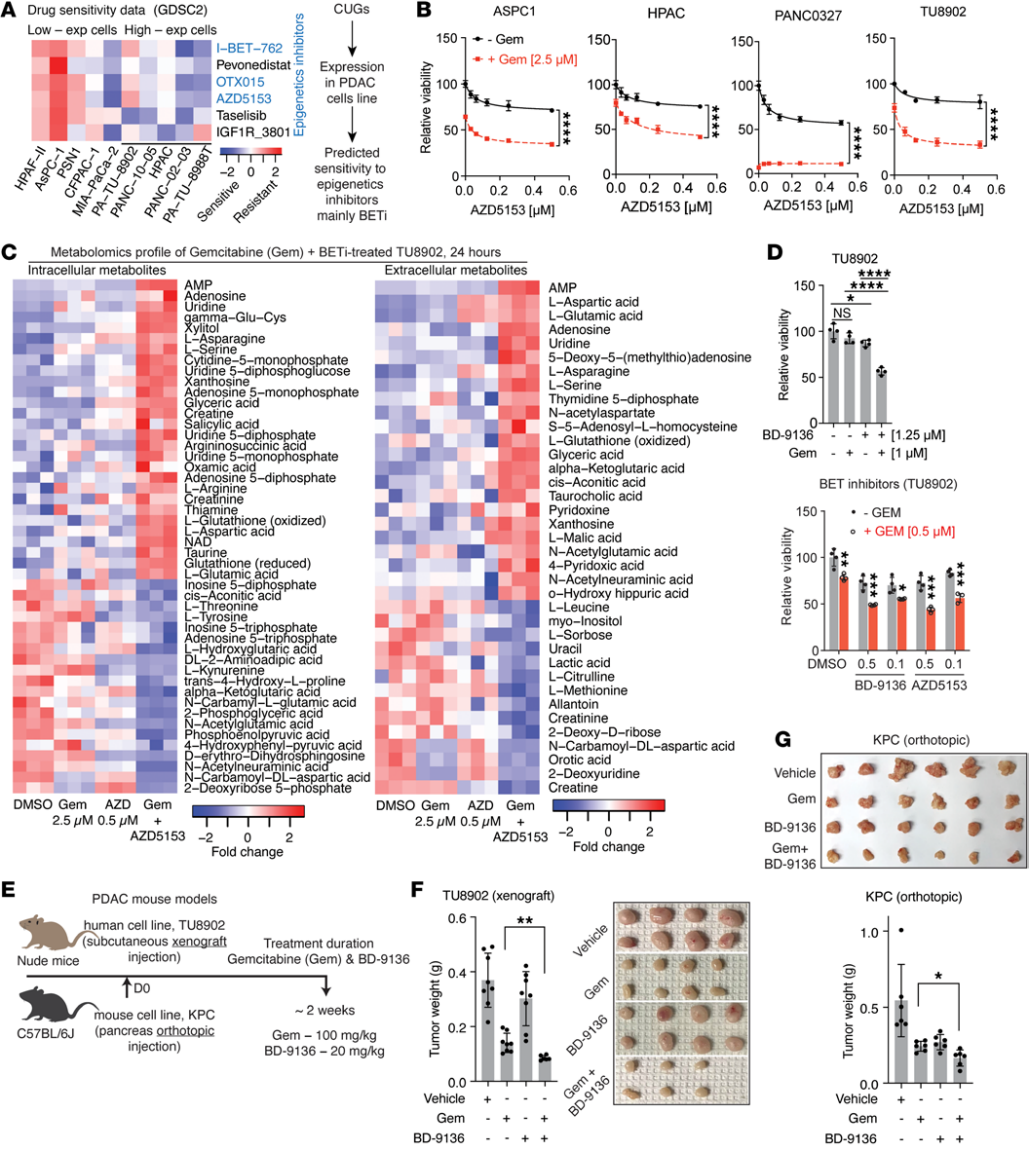
Expression of high-priority genes predicts therapy response. (**A**) Heatmap showing the sensitivity of PDAC cell lines expressing high-priority genes to compounds tested in the Genomics of Drug Sensitivity in Cancer (GDSC2) data. In blue font are epigenetics drugs. EXP, expressing. (**B**) Viability assay of PDAC cell lines treated with BETi AZD5153 alone or in combination with gemcitabine (gem) for 72 hours, in quadruplicate. BETi, bromodomain and extraterminal motif (BET) protein inhibitor(s); DMSO, dimethyl sulfoxide. (**C**) Heatmaps showing the metabolomics profile of lysate (intracellular) and culture media (extracellular) extracts from human PDAC cell TU8902 treated with gemcitabine, AZD5153, or the combination, for 24 hours, in triplicate. AMP, adenosine monophosphate; NAD, nicotinamide adenine dinucleotide. (**D**) Viability assay of TU8902 treated with gemcitabine, BD-9136 (a bromodomain-containing protein 4 degrader), or both, for 48 hours. Represents more than 2 independent experiments. Below: Treatment with BD-9136 or AZD5153 alone or in combination with gemcitabine for 48 hours, in quadruplicate. (**E**) Workflow of the mouse tumor experiments. Mice were treated with gemcitabine at 100 mg/kg body weight 2 times per week and with BD-9136 at 20 mg/kg body weight 5 times per week. (**F**) Tumor weight of subcutaneously implanted TU8902 (*n* = 4 mice per group, injected on both flanks; *n* = 8 tumors per group except combination arm, *n* = 6). Right: Image depicting the size of tumors harvested at the end of the experiment. (**G**) Image depicting the size of KPC 7940b pancreatic orthotopic tumors following the indicated treatments (*n* = 6 mice per group). Below: Bar graph showing tumor weights. Statistical comparison of the indicated groups (**F** and **G**) was by 2-tailed *t* test with Welch’s correction. Comparison by 1-way ANOVA with Tukey’s post hoc correction was not statistically significant. Comparisons for **B** and **D** (below) were by unpaired *t* test and **D** was by 1-way ANOVA with Tukey’s post hoc test. *P < 0.05; **P < 0.01; ***P < 0.001; ****P < 0.0001 (**B**, **D**, **F**, and **G**).

To predict therapies that may be effective against PDAC, we took advantage of the cancer drug response data from the Genomics of Drug Sensitivity in Cancer (GDSC) study (25). We reasoned that PDAC cell lines that express a high level of the high-priority targets from CRISPR/Cas9 and shRNA screens would likely respond to a similar set of inhibitors. Accordingly, we stratified PDAC cell lines in the cancer cell line encyclopaedia (CCLE) (26) and GSE57083 datasets (Supplemental Figure 9A) based on the gene interference screen–derived priority targets. The cell lines PA-TU-8902, PA-TU-8988T, PANC1, and PANC-03-27 expressed several of the priority targets at a high level (i.e., the high-expressing cells), whereas others like AsPC-1, PSN1, CFPAC-1, and HPAF-11 were low-expressing cells. GDSC data showed that these cells differed in their sensitivity to various inhibitors (Figure 7A and Supplemental Figure 9B). We observed that several epigenetic inhibitors, notably the bromodomain and extraterminal motif (BET) protein inhibitors (BETis) I-BET-762, OTX015, and AZD5153 emerged as effective in high-expressing cell lines. PA-TU-8902 (or simply TU8902) was the exception, indicating a potential resistance (Figure 7A).

We performed experiments with BETis to demonstrate proof of concept with respect to harnessing epigenetic modulators for pancreatic cancer therapy. BETis act by repressing transcription and cell cycle (27, 28), which are core changes associated with the CUGs in pancreatic tumors (shown in Figure 1E and Figure 2, A and H) and cell cycle is altered in PDAC cell lines (29–32). We proceeded to testing the combination of BETi with gemcitabine chemotherapy as a potential strategy to overcome therapy resistance. BETi AZD5153, a BRD4 inhibitor (33), synergized with gemcitabine to suppress cell viability in all tested cell lines (Figure 7B). Metabolomics showed that the combination treatment disrupted the intracellular and extracellular pool of amino acids, notably glutamine, glutamate, aspartate, asparagine, and serine (Figure 7C and Supplemental Figure 9C). We tested BD-9136 (a BRD4-selective degrader) (34) and observed that it synergized with gemcitabine to suppress PA-TU-8902 cell growth in vitro and in a subcutaneous xenograft (Figure 7, D–F), although staining for proliferation marker Ki67 did not reveal a clear change (Supplemental Figure 9D). The combination of BD-9136 and gemcitabine also suppressed the in vitro growth of a mouse oncogenic KRAS (KPC) PDAC cell line as well as pancreatic orthotopic tumor growth (Figure 7, E and G, and Supplemental Figure 9E). These data implicate epigenetic inhibitors as potentially effective therapies in a subset of pancreatic cancer.

## Discussion

We have leveraged a variety of published datasets to define the most consistently differential genes in human PDAC, thus providing a resource to guide mechanistic studies on this deadly cancer. The consistent genes can be expected to be markedly high (in terms of CUGs) or low (in terms of CDGs) in any comparison of PDAC tissues with nontumoral pancreas. For the majority of the genes, their function in PDAC has not been defined, thus highlighting untapped opportunities for new discoveries. Where previously studied, many of the genes were proposed as therapeutic targets or biomarkers in PDAC and their described expression pattern are in line with our findings. Accordingly, we believe that the little-to-not-yet-studied genes found in our analysis are likely important. Moreover, several genes previously identified as tumor/stroma specific or signatures of basal-like and classical PDAC subtypes also emerged in our analyses. These findings support the notion that the consistent genes are worthy of further studies in PDAC.

It is noteworthy that many known genes in cancer, e.g., *HIF1A*, *SLC16A3*, *EPCAM*, and *SOX9*, were consistent, albeit in fewer datasets and so did not meet our inclusion criteria. In addition, probe identification or name issue may have caused some genes, e.g., *KRT17* and *INHBA*, to fail our consistency test. Thus, our estimate of approximately 4,000 consistent genes (i.e., CUGs and CDGs combined) may seem numerous at face value, but is conservative and underlies the molecular complexity of PDAC. We recommend that when the goal is to determine individual overrepresented pathways to investigate in experimental contexts, pathway enrichment and GO tools (35) should be applied to enable the identification of a set of related genes.

We observed that some genes that are traditionally considered high in cancer are not tumor-specific. For example, core glycolysis genes such as *ALDOA*, *GAPDH*, *ENO1*, and *PKM2* are high in both tumor and immune cells. However, tumor nonspecificity may not render these genes less important, especially considering their correlation with proliferation, metastasis, and OS. Thus, our identification of tumor-specific and non–tumor-specific genes could facilitate hypothesis generation to test tumor-intrinsic versus stromal or immune drivers of PDAC.

By stratifying tumors using signatures of basal-like and classical subtypes from previous studies (10), we establish that PDAC may not easily cluster into 2 subtypes. Our data suggest that there possibly exists 1 other subtype that is neither basal-like nor classical or combines the features of both subtypes. This “non-basal-like non-classical” tumor subset expresses core signatures such as high *TMPRSS4*, *SLC6A14*, *IFI27*, *CST1*, and *STYK1*. Notably, *IFI27* was a strong marker of the epithelial cell compartment in our analysis and has been implicated in metastasis (36, 37). Understanding genes that are differentially expressed in PDAC but did not fall into basal-like or classical subtype signature could uncover new mechanisms underlying PDAC.

Our data make clear that PDAC is molecularly complex and involves multiple pathways. Although “cell cycle” and “glycolysis” often ranked at the top, changes in PDAC cuts across other pathways, including “focal adhesion,” “p53,” “ECM-receptor alterations,” and “pathway in cancer.” However, the high expression of glycolysis and cell cycle/division pathways was so consistent that it seems plausible that these changes are central to the origin or progression of PDAC. This notion is consistent with a prior study, which found that the genomic rearrangements and changes in cell cycle likely occur earlier in PDAC (18). But, knowledge gaps still exist; several cell cycle genes we identified have never been studied in PDAC. On the part of glycolysis, despite the long-standing appreciation that glucose metabolism is altered in PDAC, the role of specific glycolytic enzymes or their isoforms e.g., *HK*s, *PFKP*, and *ENO2*, or the therapeutic prospects of inhibiting these genes is largely unclear. Our observation that these glycolysis genes do not necessarily show tumor-specific expression patterns could guide future mechanistic studies on how best to therapeutically exploit glucose metabolism in PDAC. We also identified *KRAS* among the priority genes. While targeting KRAS mutation was ineffective in a prior clinical trial (38), a new KRAS inhibitor has emerged and been shown to be effective in PDAC (39–41). The consistent emergence of *KRAS*, coupled with experimental evidence of its role in tumorigenesis in mice (6, 7), suggests that studies should continue to explore better ways to target the Ras pathway. Other processes such as “proteasomes” and “spliceosomes” contained consistent genes that showed essentiality across high-throughput gene interference screens (21, 22, 24).

Lastly, we showed that the CUGs could be used to predict therapy, which further broadens the opportunity to identify effective treatment options against PDAC. However, despite the enormity of data we used, this study has limitations. Specifically, although some of the priority genes have been functionally characterized in PDAC, mechanistic studies are needed to determine whether all the priority genes are functionally relevant. In addition, the datasets used did not have the same number of genes. This implies that since our inclusion cutoff was consistent expression in 4 out of 5 datasets, any gene that was not present in 2 datasets was excluded even if consistent in the other remaining 3 datasets. Therefore, we do not intend to suggest that these are the only important genes in PDAC. Furthermore, the selection of an epigenetic modulator for the mouse study was to demonstrate proof of concept. We did not rule out whether other more potent inhibitors exist. In conclusion, we have performed the most comprehensive multidimensional analysis of gene expression pattern in PDAC to date. Our results offer a knowledge base and resource for further high-confidence basic, translational, and clinical studies. It is expected to revolutionize future understanding of true drivers of PDAC and facilitate the application of such insight in improving patient care.

## Methods

Further details can be found in the Supplemental Methods.

### Sex as a biological variable.

Our analysis of the tumor datasets included samples derived from both sexes, as our goal was to identify consistent genes in pancreatic tumors. We exclusively used female mice because of their more docile nature and therefore less variable results. We expect the results to be relevant in male mice as well.

### Subcutaneous xenograft tumor implantation.

For mouse tumor xenografts, approximately 6-week-old female athymic nude mice NU/J (stock 002019, The Jackson Laboratory) were maintained in the facilities of the Unit for Laboratory Animal Medicine (ULAM) at the University of Michigan under specific pathogen–free conditions. On the day of xenograft tumor injection, the PA-TU-8902 cell line was harvested from culture plates according to normal cell culture procedures. The cells were counted, washed once with PBS, and resuspended in a 1:1 solution of serum-free DMEM (Gibco, 11965-092) and Matrigel (Corning, 354234). Mice were subcutaneously injected on both flanks with 0.5 × 10^6^ cells in 100 μL volume. When tumors became palpable, mice were randomized into the 4 treatment groups: vehicle, gemcitabine, BD-9136, and gemcitabine plus BD-9136, following the same dosing regimen used for orthotopic experiment. Tumor size was monitored twice per week using a digital caliper and tumor volume was calculated as *V* = 0.5 × (length × width^2^). At the endpoint, mice were euthanized, and the tumors harvested, weighed, and processed for further analysis.

### Mouse pancreatic orthotopic tumor model.

To establish the orthotopic model, 50,000 KPC cells were injected into the pancreas of wild-type female C57BL/6J mice (~8 weeks old) following laparoscopy. The cell lines were suspended at 1:1 ratio of serum-free DMEM and Matrigel and injected in a 50 μL final volume. Ten days after injection, the mice were randomized into experimental and control groups (*n* = 6 mice) based on body weight. Experimental groups consisted of gemcitabine (100 mg/kg body weight twice per week), BRD4-selective degrader BD-9136 (20 mg/kg body weight 5 days per week), or both. Vehicle consisted of 20% PEG400, 6% Cremophor EL, and approximately 74% PBS solution. Treatments were administered by intraperitoneal injection. At the endpoint, tumor weights were obtained and tissues extracted for further processing.

### Identification of the consistent genes.

The following 5 human PDAC microarray datasets were used for the initial identification of the consistent genes: GSE71729 (*n* = 46 normal pancreas vs. 145 tumor tissues), GSE62452 (*n* = 61 nontumoral vs. 69 tumor tissues), GSE28735 (*n* = 45 nontumoral vs. 45 tumor tissues), GSE16515 (*n* = 16 normal vs. 36 tumor tissues), and GSE15471 (*n* = 36 nontumoral vs. 36 tumor tissues). These datasets were obtained from the NCBI GEO (https://www.ncbi.nlm.nih.gov/geo/). The tumors were mainly from surgery, as indicated in the original publication of the datasets. Of note, these datasets were not combined, but rather analyzed separately. For each dataset, the differentially expressed genes in pancreatic tumors compared with nontumoral control samples were determined using the *limma* package (v3.38.3) in R software (v3.5.2; https://www.r-project.org/) after quantile normalization. Where there are multiple probes identifying a particular gene, we obtained the average for that gene prior to analysis. We used an adjusted *P* value of less than 0.05 for selecting differentially expressed genes per dataset. Thereafter, we overlapped the differential genes across the 5 datasets to identify genes that were similarly up- or downregulated in at least 4 datasets. Genes that met the adjusted *P*-value cutoff, and were upregulated in at least 4 of the 5 datasets, were called consistently upregulated genes (CUGs). Genes that were expressed at low levels in at least 4 datasets were called consistently downregulated genes (CDGs). Further details are in the Supplemental Methods.

### KM OS, Cox regression, and prognostic outcome analyses.

KM OS was performed for each gene in 4 patient cohorts: TCGA (*n* = 146 tumors), GSE71729 (*n* = 125 primary tumors), Puleo (*n* = 288 tumors), and ICGC-AU (*n* = 267 tumors). For TCGA, we selected the samples that were used for survival analysis in the original article on PDAC (4). For the other datasets, we included all samples for which there are accompanying survival data. For each gene in a patient cohort, we derived its median expression value and used that to rank the samples into “high” and “low” groups for that gene. We then performed KM OS analysis with a log-rank test. Univariate Cox proportional hazards regression analysis was also performed with the same sample size as used for KM except that the gene expression was set as continuous variable and the associated *P* values were derived using Wald statistics. Both the KM and Cox regression analysis were performed using the *survival* package (v2.43-3) in R, with *P* less than 0.05 considered statistically significant. We placed greater emphasis on genes that statistically predicted outcome in 2 or more cohorts. We also compared the consistent gene expression in tumors of patients with “bad” prognosis (shorter survival time) versus “good” prognosis (longer survival time) using the microarray dataset GSE42952 (tumors: *n* = 6 good prognosis vs. *n* = 6 bad prognosis). The data were analyzed following the same procedure as in the 5 microarrays used to identify CUGs/CDGs.

### Statistics.

Details about the analyzed or plotted samples are described above and presented in figure legends where applicable. Survival plots were generated with the *survminer* package (v0.4.3). Additional R packages used were *pheatmap* (v1.0.12) and gplots (v3.0.1) for heatmaps, *DESeq2* (v1.43.5) for RNA-seq data, *wordcloud* (v2.6) for generating word clouds, and *VennDiagram* (v1.7.3) for Venn diagrams. Network visualization and disease ontology analysis were performed with enrichplot (v1.6.1) and DOSE (v3.12.0) in R (v3.6.2). We determined which CUGs or CDGs are well studied or uncharacterized/novel in PDAC by searching the PubMed portal using each gene’s symbol. The search terms were the gene symbol and “pancreatic” or “pancreatic cancer” and genes that returned between 0 and 4 relevant results were considered novel.

### Study approval.

All animal studies were performed in accordance with the guidelines and approval (protocol no. PRO00008877) of the Institutional Animal Care and Use Committee (IACUC) of the University of Michigan.

### Data availability.

The underlying data for the presented figures are available in the Supporting Data Values file. The gene lists are provided as Supplemental Tables 1–13. The gene datasets used in this study are publicly available under the indicated accession numbers along with the clinical data where applicable. The microarrays were obtained from NCBI GEO or ArrayExpress databases. The ICGC-AU microarray data were downloaded from the ICGC portal along with the associated clinical data and had no embargo (March, 2020). The cancer essential gene data from the Project Achilles, Project Drive, and GECKO screens were downloaded from the DepMap Public 20Q1, while “03_scaledbayesianfactor” data from the Behan et al. (22) CRISPR screen were downloaded from the BioGRID ORCS portal. Drug response data were accessed via the GDSC portal. The URLs of the respective portals are listed under “URLs” in the Supplemental Methods. The R code/script used for deriving the consistent genes is provided as Supplemental Dataset 1.

## Author contributions

ZCN conceived and designed the study, performed data analyses and experiments, and wrote the manuscript. HMG, MN, VC, DK, SBK, WKML, IL, NS, PS, AA, and LZ provided sample and data analysis. REM, JH, and BH performed experiments. SW, MPM, and CAL provided materials/reagents and designed experiments. MPM and CAL supervised the project. All authors read and approved the final manuscript.

## Supplementary Material

Supplemental data

Supplemental tables 1-13

Supporting data values

## Version 1 01/07/2025

In-Press Preview

## Version 2 02/24/2025

Electronic publication
