# Supplementary material for: Multidimensional analyses identify genes of high priority for pancreatic cancer research
Source: JCI Insight. 2025 Jan 7;10(4):e174264. doi: 10.1172/jci.insight.174264 (PMC11949049; doi:10.1172/jci.insight.174264)
Supplement: Supplemental data [file jciinsight-10-174264-s247.pdf]

Multi-dimensional analyses identify genes of high priority for pancreatic cancer research

Supplemental Figures and Methods

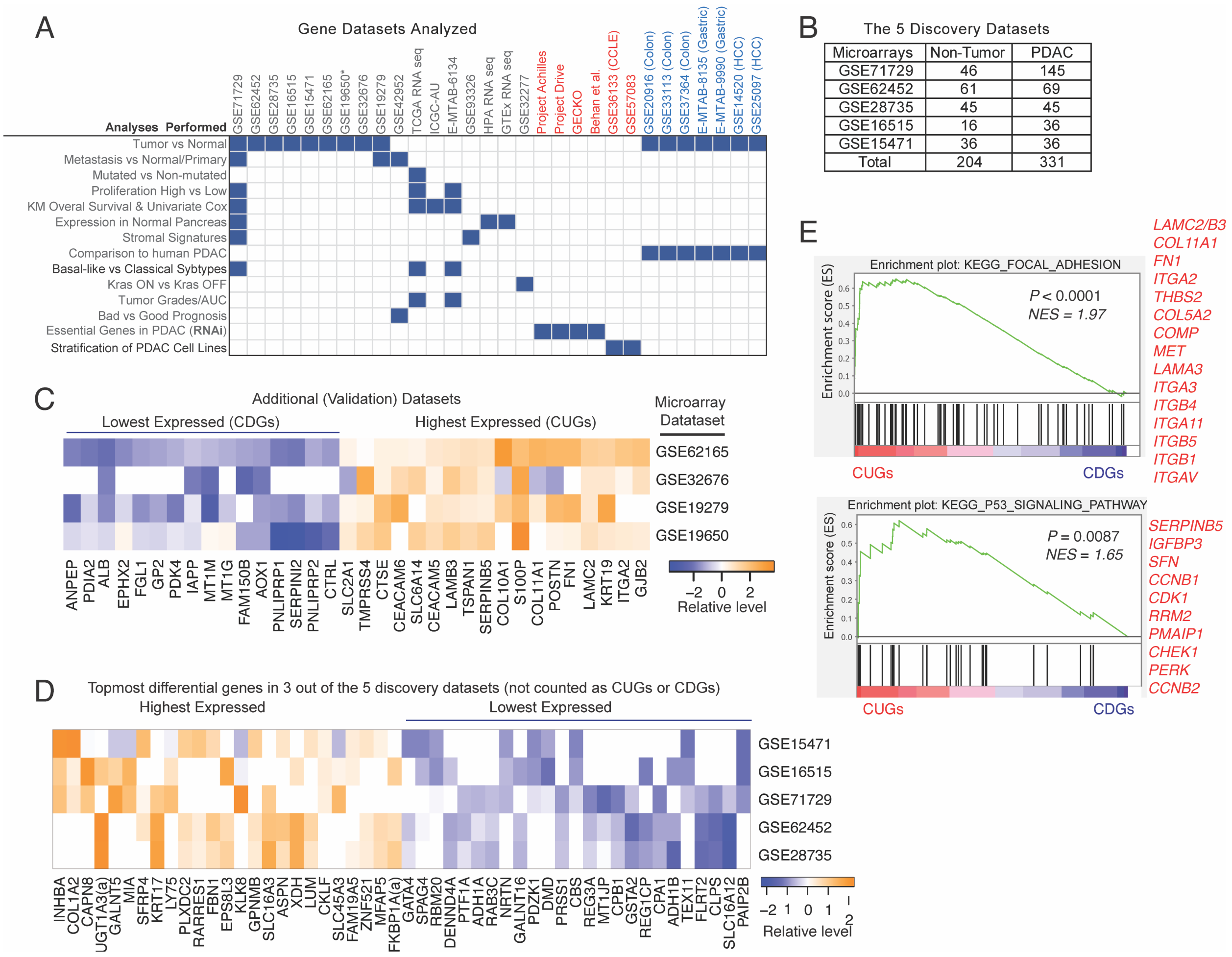

Supplemental Figure 1. Consistently expressed genes in human PDAC tissues

(**A**) Gene datasets analyzed in the quest for consistent genes, priority targets and drug response predictors in PDAC. In blue are datasets of other gastrointestinal tumors, specifically colon, gastric, and liver cancers.

(**B**) The five "discovery" microarray datasets of pancreatic ductal adenocarcinoma (PDAC) used for the identification of the consistently upregulated genes (CUGs) and consistently downregulated genes (CDGs). Genes were defined as 'consistent' if upregulated or downregulated with an adjusted *P*<0.05 in at least 4 of the 5 datasets.

(**C**) Heatmap showing that the additional microarray datasets reproducibly depicting the topmost consistent genes derived from the 5 'discovery' datasets.

(**D**) Topmost up- or downregulated genes expressed in 3 of the 5 “discovery” datasets. In **C-D**, orange – upregulated; blue – downregulated; blank/white – no data or not changed in the corresponding dataset. UGT1A3(a): named across the datasets as *UGT1A3///UGT1A1///UGT1A4///UGT1A9///UGT1A5///UGT1A6///UGT1A7///UGT1A8///UGT1A10*
(**E**) Gene set enrichment analysis (GSEA) plots showing additional pathway enrichment in PDAC as derived with the consistent genes. NES – Normalized Enrichment Score.

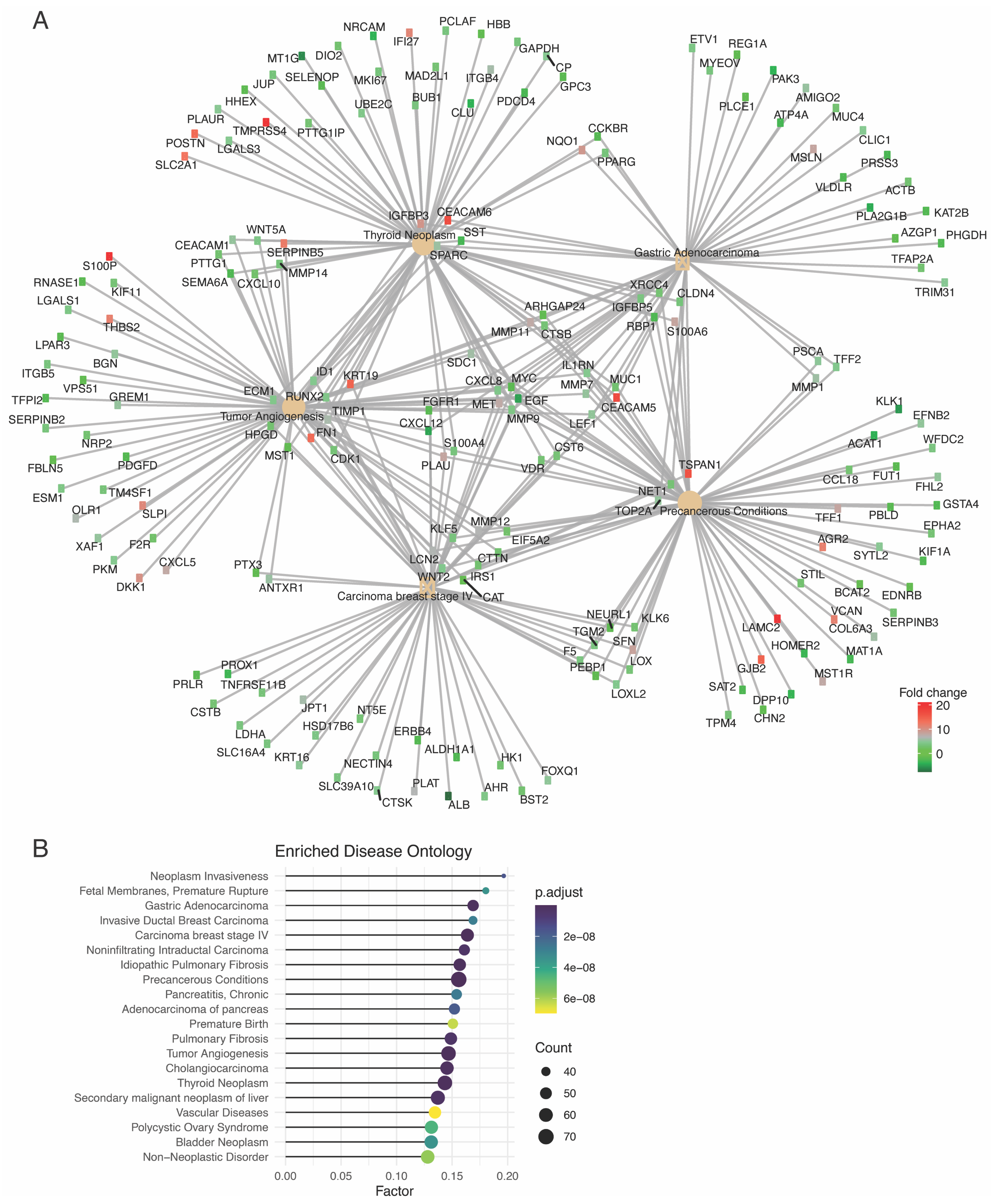

Supplemental Figure 2. Network and pathway annotation analyses of the consistent genes

(**A**) Interactome network analysis derived using the top 500 CUGs and 500 CDGs.

(**B**) Enrichment plot showing the disease ontology associated with the top 500 CUGs and 500 CDGs.

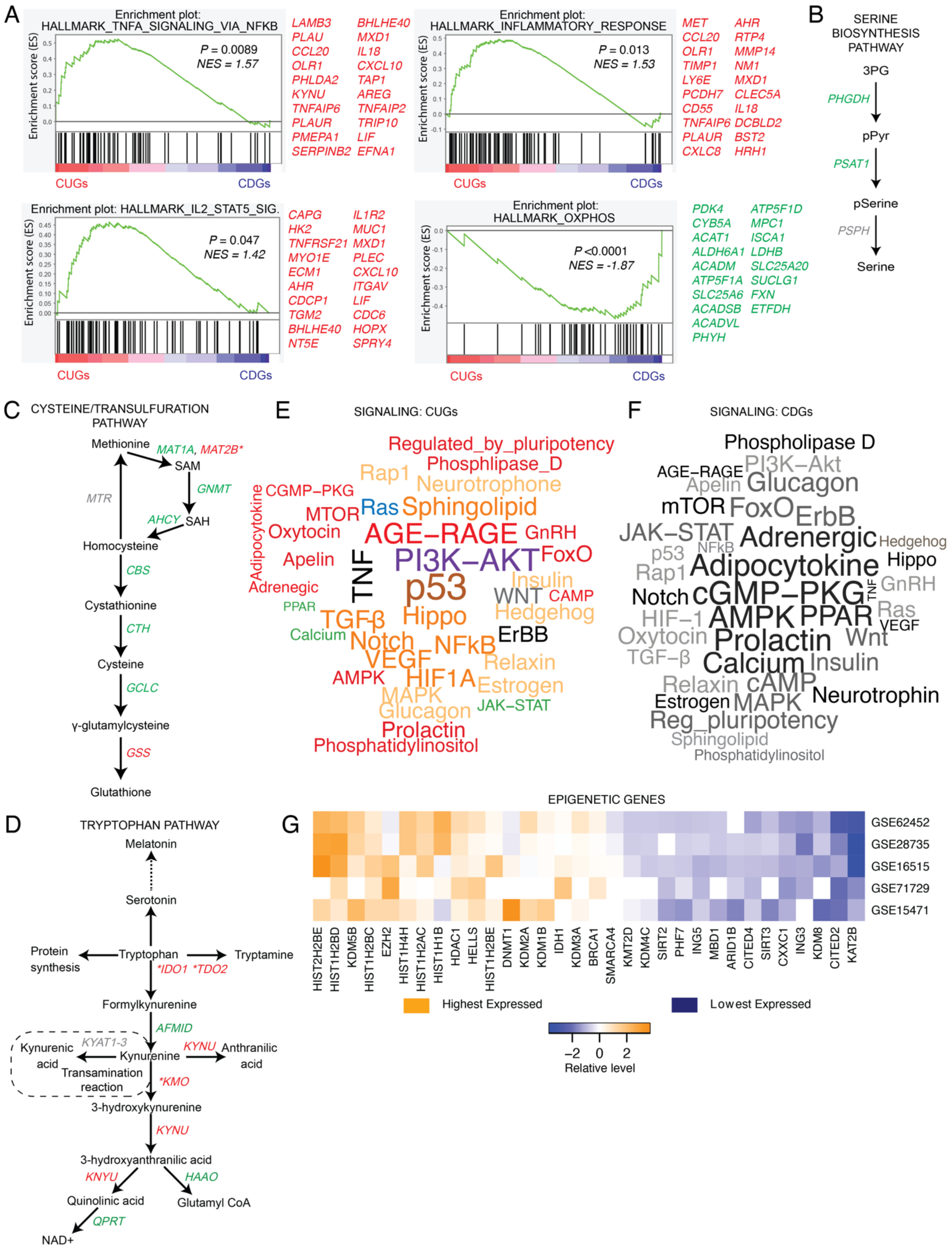

Supplemental Figure 3. Consistent genes are present in multiple molecular pathways

(**A**) GSEA plots showing upregulated Hallmark TNFA (tumor necrosis factor) signaling, inflammatory response, IL2 (interleukin 2)-STAT5 sig. (signaling) and downregulated oxidative

phosphorylation (OXPHOS) in PDAC based on the consistent genes. NES – Normalized Enrichment Score.
(**B**) Serine pathway genes are consistently downregulated in PDAC.
(**C**) Schematics of the cysteine/transsulfuration and (**D**) tryptophan pathways.
(**E**) Word cloud showing the signaling processes associated with the CUGs and (**F**) the CDGs.
(**G**) Heatmap showing the most consistently expressed epigenetics genes (top 20 up/downregulated) across the indicated five PDAC datasets. Genes in gray in **B-D** were not changed at adjusted $P<0.05$ to meet inclusion criteria.

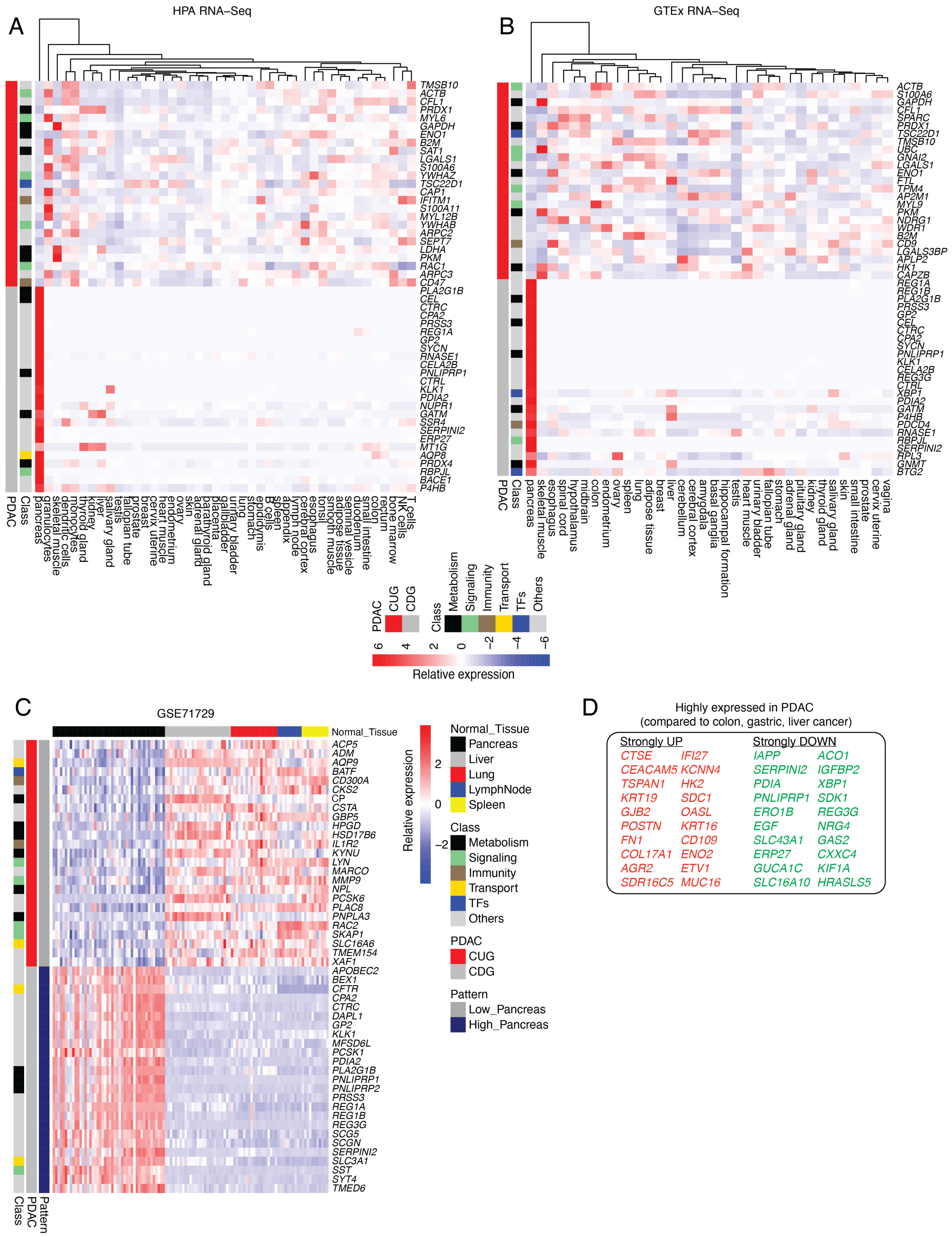

Supplemental Figure 4. CUGs and CDGs are low and high, respectively, in normal pancreas tissue compared to other tissues

(**A**) Heatmap showing CUGs that are the top lowly expressed or CDGs that are the top highly expressed in normal pancreas relative to other tissues in the Human Protein Atlas RNA-seq data, and (**B**) the genotype-tissue expression (GTEx) project data.
(**C**) Heatmap showing the topmost CUGs lowly expressed, or CDGs highly expressed, in normal pancreas compared with normal liver, lymph, lung tissues in the GSE71729/Moffitt dataset (P<0.01).
(**D**) Highly specific genes mostly ranked within the top 100 upregulated or downregulated in PDAC in at least in 4 of 5 datasets but not similarly (or less strongly) expressed in datasets of other gastrointestinal cancers analyzed (colon, gastric or liver cancer, adjusted P<0.05).

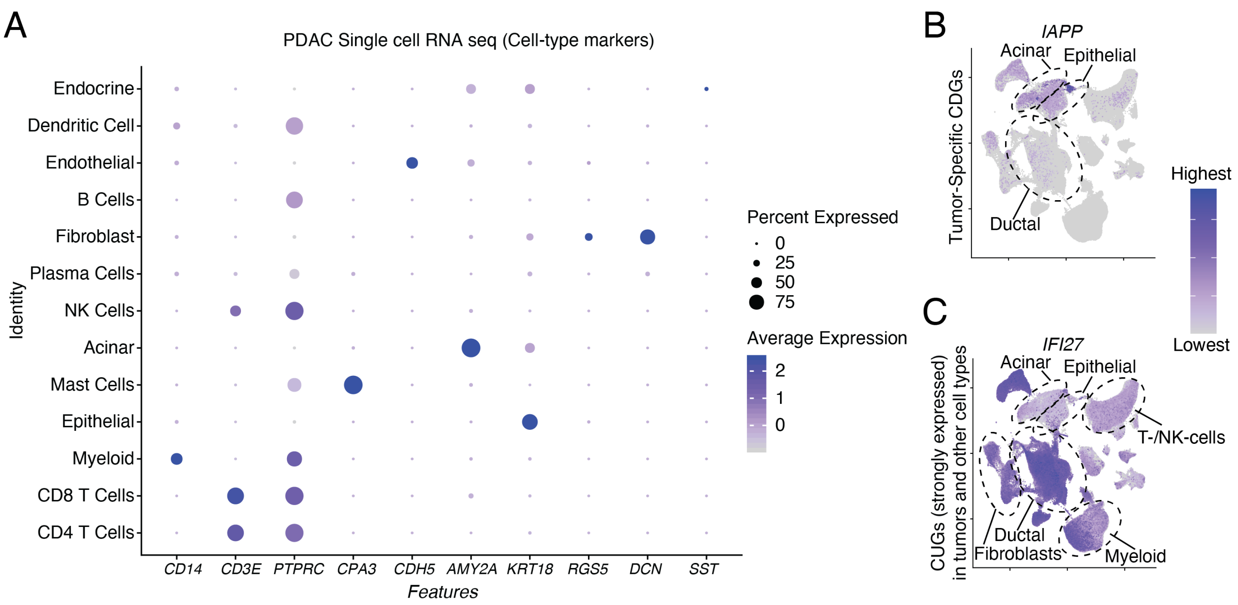

Supplemental Figure 5. PDAC scRNA sequencing data and gene expression

(**A**) Dot plot showing gene markers of various cell populations in the PDAC single cell RNA sequencing data used to validate tumor-specific and microenvironmental cell expression pattern of CUGs and CDGs. Sample size $n = 61$ primary tumors.

(**B**) Uniform manifold approximation and projection (UMAP) depicting the expression of *IAPP*, a CDG, and (**C**) CUG *IFI27* (identified as tumor-specific in microarray/RNA-seq but expressed in multiple cell populations).

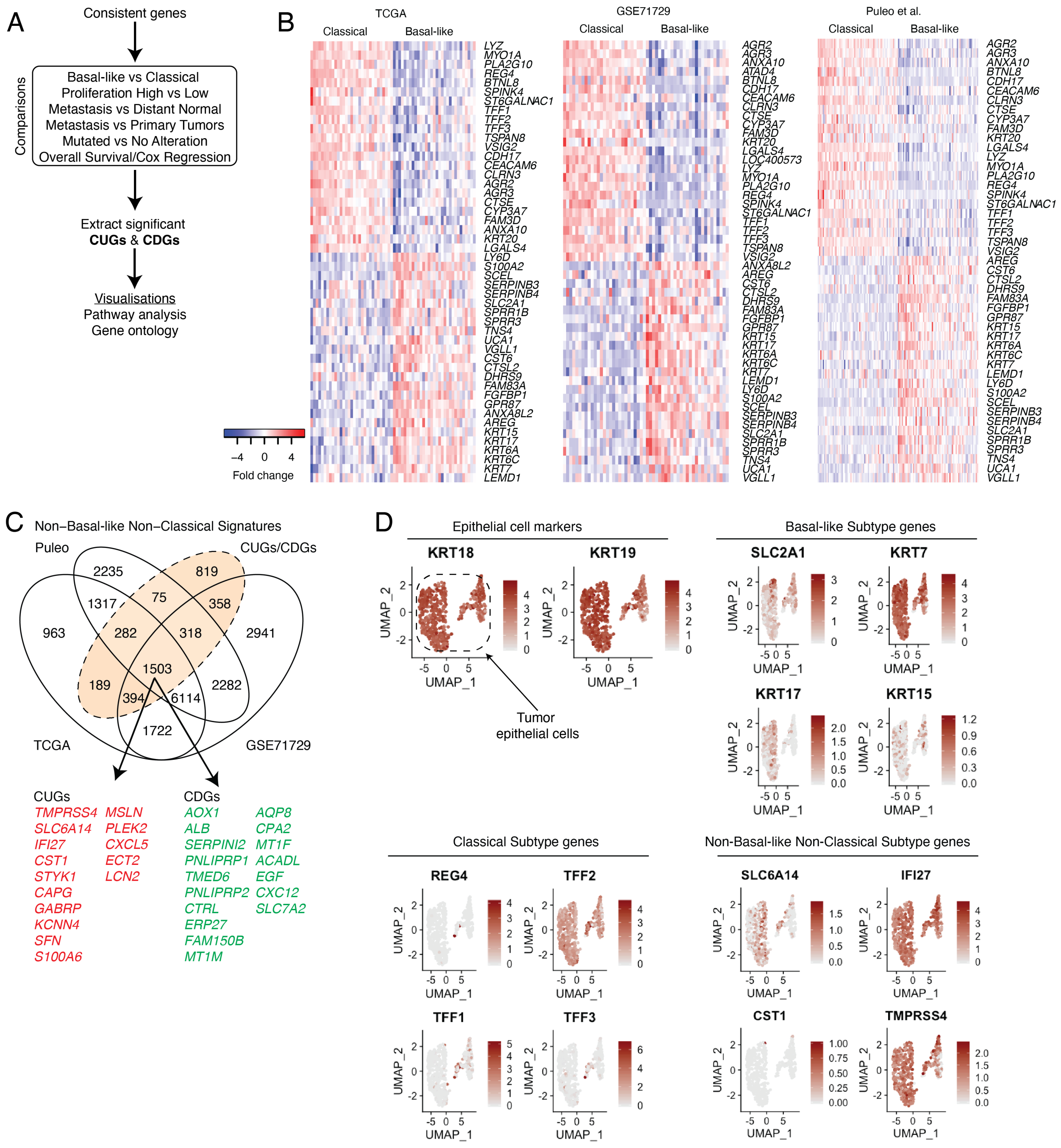

Supplemental Figure 6. Consistent genes correlate with tumor subtypes and features

(**A**) Workflow of the analysis performed to identify association of the consistent genes with clinicopathological variables. Tumor stratification was performed with TCGA (*n* = 150 samples), GSE71729 (*n* = 125), and Puleo (*n* = 309) cohorts; mutation analysis was with TCGA, while OS and Cox regression was with TCGA (*n* = 146), GSE71729 (*n* = 125), Puleo (*n* = 288) and ICGC-AU (*n* = 269) cohorts.

(**B**) Heatmap showing the stratification of three patient cohorts into classical and basal-like subtypes. Stratification was done with the top 50 gene signatures published by Moffitt et al (10), most of which were also present in TCGA and Puleo et al datasets.
(**C**) Venn diagram showing the overlap of CUGs and CDGs with genes that were not changed (P>0.05) in the comparison of basal-like versus classical tumors in TCGA, Puleo et al. and GSE71729 (Moffitt) tumors. Below, the topmost CUGs and CDGs that were not differentially expressed in basal-like or classical tumors in all three datasets are highlighted.
(**D**) Representative patient scRNA-seq data showing the epithelial cell population (marked by KRT18/19) and the expression of genes identified in basal-like, classical, and 'non-basal-like non-classical' subtypes of PDAC.

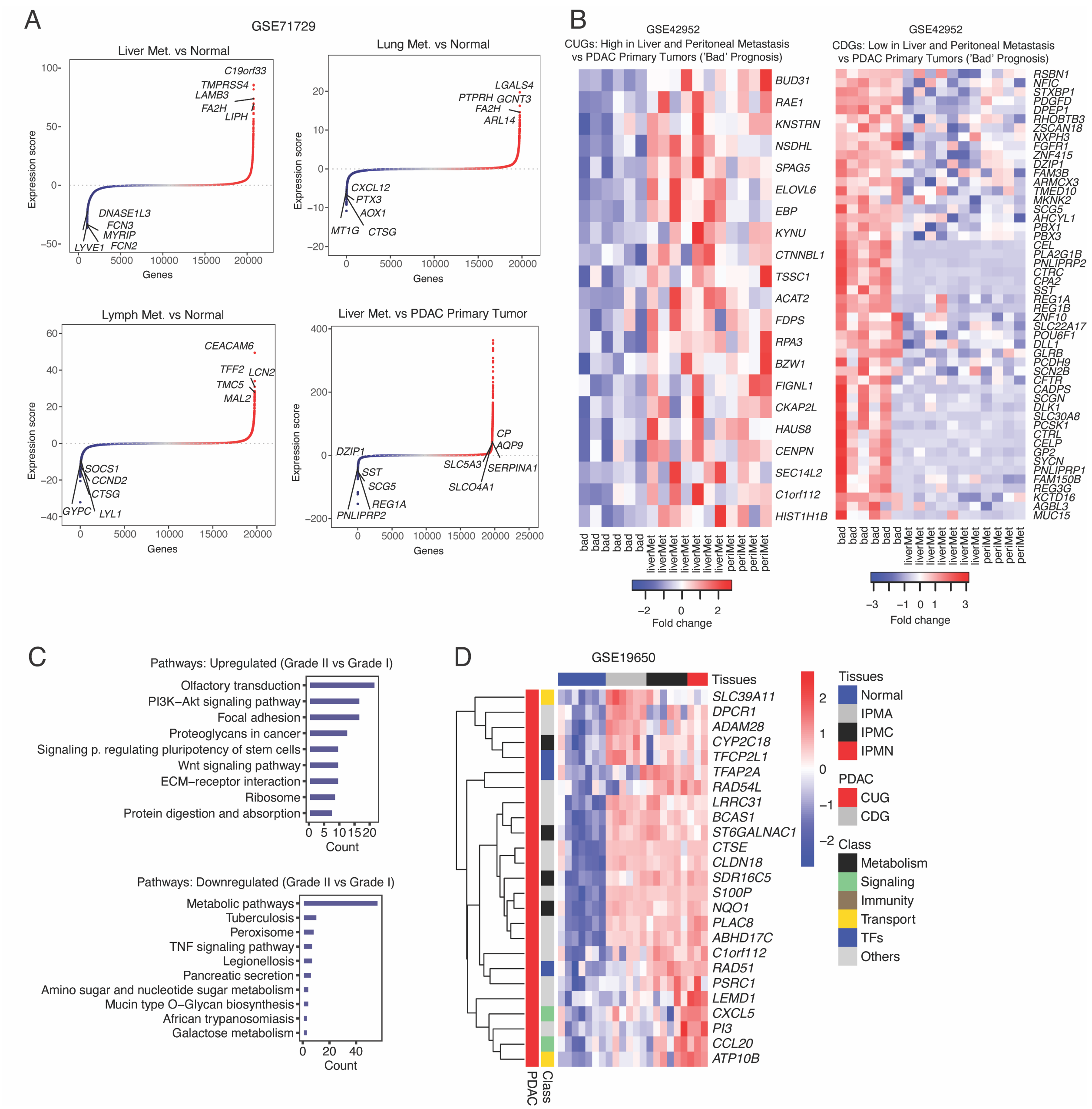

Supplemental Figure 7. Consistent genes correlate genes with metastasis and tumor grade

(**A**) Plots showing CUGs and CDGs in the topmost genes in metastasized versus normal primary tissues or liver metastasis compared with primary PDAC tumors in the dataset GSE71729.

(**B**) Heatmap showing CUGs that are high or CDGs that are low in metastasis compared to primary PDAC tumors ('bad' prognosis subset) in the dataset GSE42952.

(**C**) Pathways upregulated or downregulated in Grade II relative to Grade I tumors from Puleo et al dataset.

(**D**) Heatmap showing top CUGs differentially expressed in premalignant pancreatic diseases, e.g., IPMA – intraductal papillary mucinous adenoma, IPMC – intraductal papillary mucinous

carcinoma, IPMN – intraductal papillary mucinous neoplasms in the dataset GSE19650 (number of samples: normal main pancreatic duct = 7, IPMA = 6, IPMC = 6, IPMN = 3).

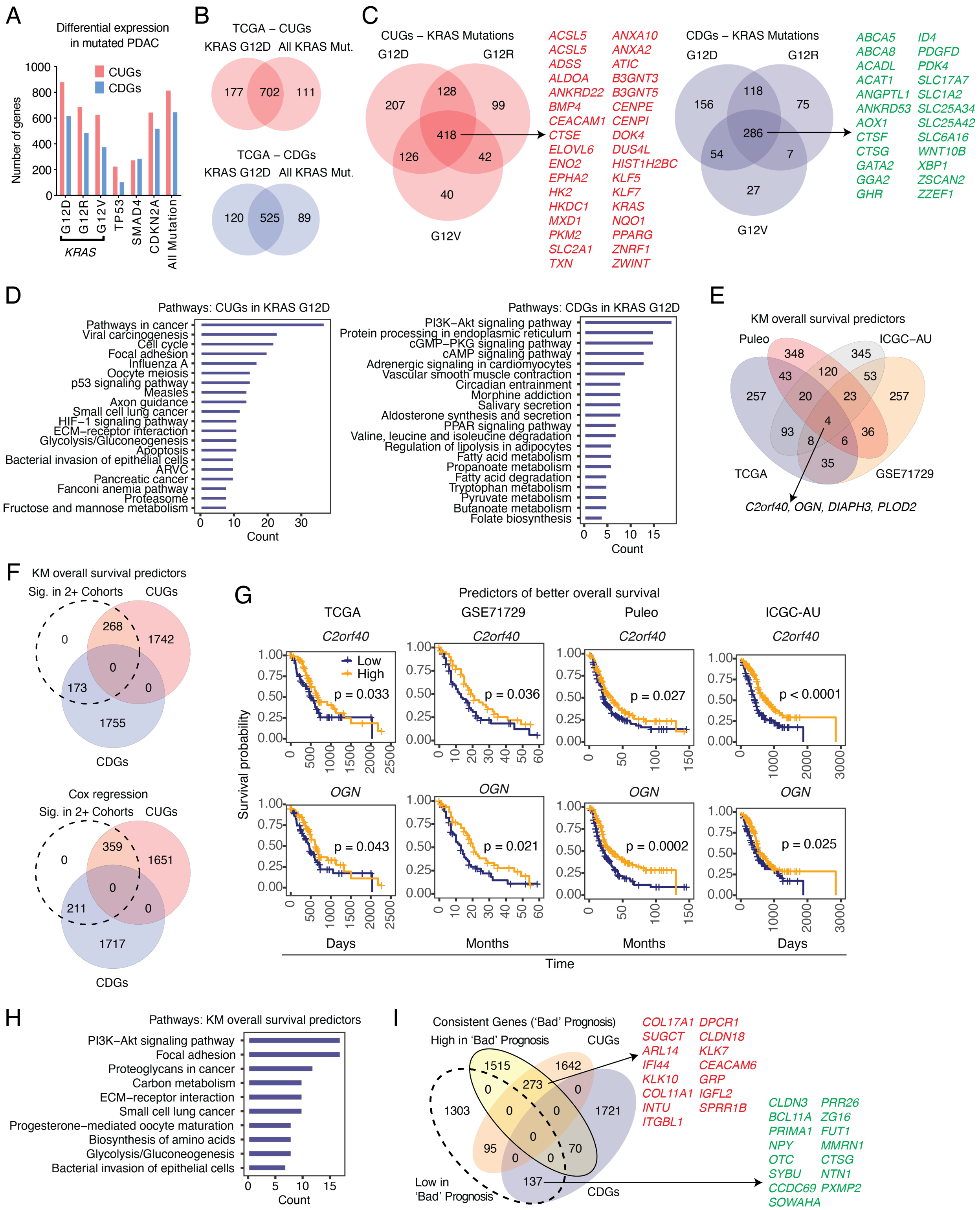

Supplemental Figure 8. Consistent genes correlate with gene mutations in PDAC and poor prognosis

(**A**) Number of differentially expressed genes in tumors with mutation versus tumors with no alteration for each of the indicated genes in TCGA data. The bars labeled “All mutation” refer to

tumors with mutation in any of *KRAS*, *TP53*, *SMAD4*, *CDKN2A* versus tumors with no recorded mutation.
(**B**) Venn Diagram showing overlap of genes differentially expressed in KRAS G12D tumors versus in tumors with “All KRAS Mut. (mutation)”.
(**C**) Venn diagram showing the overlap of GUGs and CDGs respectively expressed in various KRAS mutation tumors relative to tumors without KRAS mutation. See Supplemental File Methods for sample sizes.
(**D**) Pathways enriched by genes upregulated or downregulated in tumors in KRAS G12D mutation.
(**E**) Venn diagram showing overlaps of KM overall survival predictor genes in TCGA, GSE71729, Puleo et al and ICGC-AU cohorts.
(**F**) Venn diagram showing the number of CUGs and CDGs that predicted OS or hazard ratio (univariate Cox regression) in at least 2 out of TCGA, GSE71729, Puleo et al and ICGC-AU cohorts. Sig – significant (P<0.05).
(**G**) Kaplan-Meier (KM) overall survival plots (log-rank test, P<0.05) of genes that predicted survival in the clinical cohorts analyzed. Tumor sample size, TCGA (n = 146), Puleo (n = 288); ICGC (n = 267); GSE71729 (n = 125).
(**H**) Pathway annotation of the 441 genes that predicted overall survival in at least two of four datasets.
(**I**) Venn diagram showing the number of CUGs and CDGs that are top lowly expressed in tumors from patients that had ‘bad’ prognosis (shorter survival time, <7 months, n = 6) compared to those that had ‘good’ prognosis (longer survival time, >50 months, n = 6) based on the dataset GSE42952.

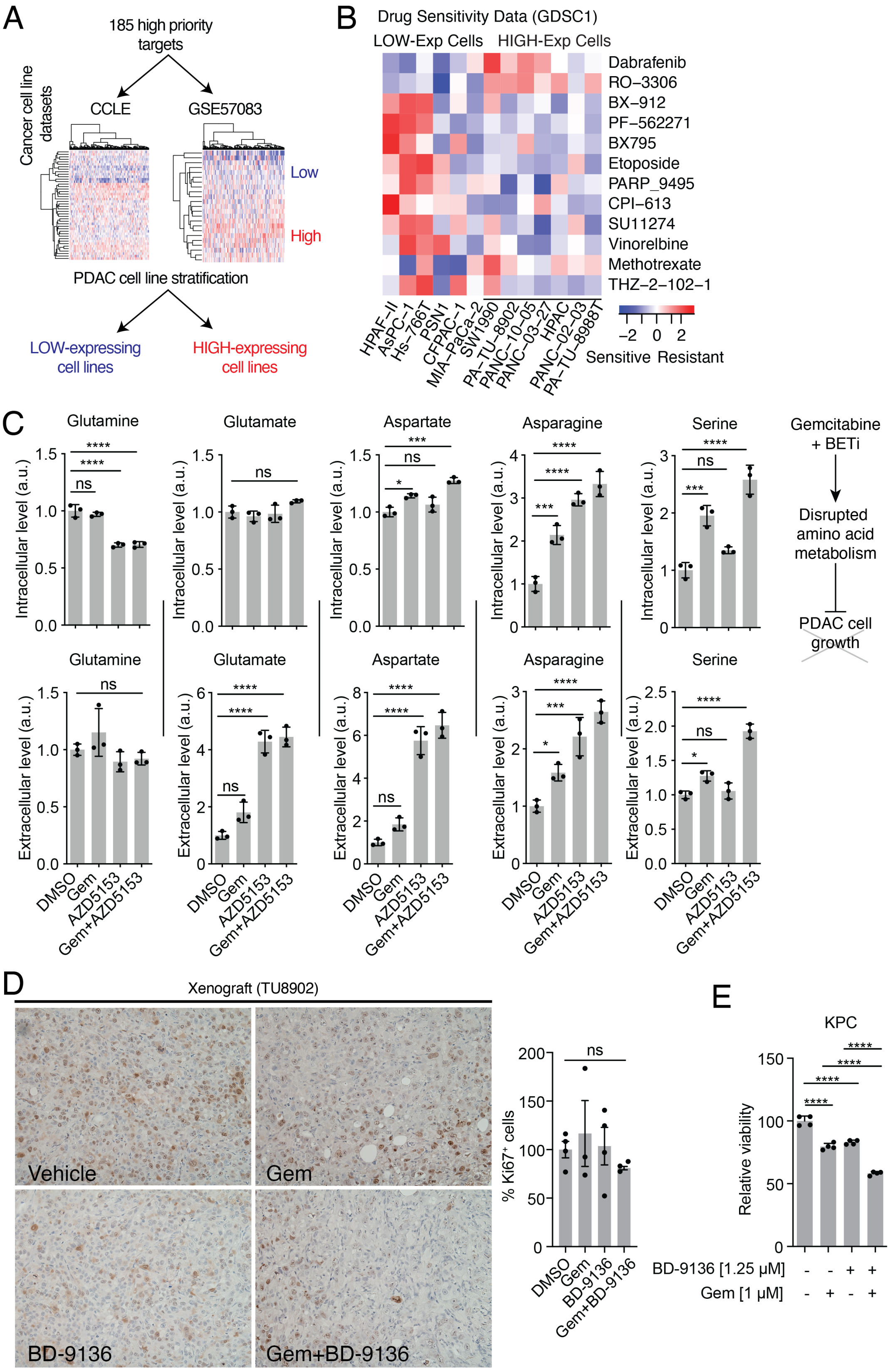

Supplemental Figure 9. Expression of high priority genes predict therapy

(**A**) Schematic illustration of the cell line stratification. The 185 high priority targets were used to stratify PDAC cells in the cancer cell line encyclopedia, CCLE (*n* = 44 cell lines) and GSE57083 datasets (*n* = 23 cell lines) into those expressing the priority genes at high and low levels, respectively.
(**B**) Heatmaps showing the sensitivity of PDAC cell lines expressing high priority genes to compounds tested in the Genomics of Drug Sensitivity in Cancer (GDSC1) data. Exp – expressing.
(**C**) Bar graphs highlighting amino acid changes in the lysate (intracellular compartment) or culture media (extracellular compartment) of the TU8902 cell lines treated with 1 μM gemcitabine (gem), 2.5 μM AZD5153 or both. These plots are an extension of the metabolomics data in Figure 7C. Data are normalized to the untreated (i.e., dimethyl sulfoxide, DMSO) control; a.u. – arbitrary unit. On the right: a schematic summary of the effect of gemcitabine and BETi on metabolism and cell growth.
(**D**) Representative micrograph (20X) of Ki-67 staining of the xenograft tumor samples derived from TU8902 cell lines. On the right: quantification of Ki-67 (proliferation marker) expression. This figure is an extension of Figure 7F.
(**E**) Viability assay of the mouse PDAC cell line KPC 7940b treated with gemcitabine, BETi BD-9136 or both for 48 h. Experiments were performed in quadruplicates. Statistical significance was tested by analysis of variance (ANOVA), **** $P<0.0001$, ns = not significance.

Methods

Identification of the consistent genes

Following the differential analysis of the PDAC datasets, we generated a matrix file containing the significantly changed consistent genes for each of the five datasets (*n* = 2010 CUGs, and *n* = 1928 CDGs in total). For some gene symbols and alias (e.g., *PKM2*), conversion to current human gene nomenclature was necessary for cross-dataset comparison. For that we used either the https://www.biotools.fr/ portal or *limma* built-in function for alias conversion. The consistent genes were ranked using the sum of 'expression score' (i.e., the product of logFC and -log *P*-value) across the five datasets. Besides the list of CUGs and CDGs (i.e., genes similarly up- or downregulated in at least four datasets at adjusted $P<0.05$), we extracted genes that showed consistent expression in at least three datasets, provided they are unchanged in the remaining two datasets (*n* = 2,722, adjusted $P<0.05$, see **Supplemental Table 1B**). These genes were only used in completing metabolic pathway drawings in cases where they are critical components of the pathway but did not meet selection cut-off of the CUGs and CDGs list described in this work. Where applicable, those genes were marked with asterisks.

Cross-validation of the consistent genes in independent datasets

For cross-validation of CUGs and CDGs expression in independent patients cohorts, we included four additional microarray datasets, namely, GSE32676 (7 non-malignant pancreas vs 42 tumor tissues, $P<0.05$), GSE62165 (13 control samples vs 118 tumors, adjusted $P<0.01$), GSE19279 (compared 3 normal vs 4 tumors, $P<0.05$), and GSE19650 – a laser capture microdissection data consisting of 7 normal pancreas vs pre-malignant tissues of invasive cancer originating in intraductal papillary-mucinous neoplasm (IPMN, n = 3), intraductal papillary-mucinous adenoma (IPMA, n = 6), and intraductal papillary-mucinous carcinoma (IPMC, n = 6), $P<0.05$). These datasets were processed and statistically compared following same methods used to derive the consistent genes. Of note, some tumor datasets used in our study have additional sample groups beyond tumor vs non-tumor description. Where those additional samples were analyzed (e.g., metastasis vs distant normal tissues in GSE71729, we followed the analysis steps used for tumor vs non-tumors). Additional microarray datasets included were E-MTAB-6134 (309 tumors) obtained from ArrayExpress portal as well as the International Cancer Genome Consortium – Australia cohort (ICGC-AU, 269 tumors). These two data were mainly used for tumor stratification, clustering and analysis of correlation with clinical parameters (see **Supplemental Figure 1A** for the dataset accession numbers). To analyze the expression of PDAC consistent genes in other gastrointestinal cancers (i.e., to determine the specificity of the genes to PDAC) we used two datasets of liver-, two of gastric- and three of colon cancers. The liver cancer datasets were GSE14520 (n = 220 non-tumor liver samples, n = 225 tumors) and GSE25097 (n = 243 non-tumor liver samples, n = 268 tumor samples). Gastric cancer datasets were E-MTAB-9990 (n = 16 non-tumors, n = 16 tumor samples) and E-MTAB-8135 (n = 10 non-tumors, n = 10 tumor samples). Colon cancer datasets were GSE20916 (n = 24 non-tumors, n = 36 tumor samples), GSE33113 (n = 6 non-tumors, n = 90 tumor samples), and GSE37364 (n = 38 non-tumors, n = 14 tumor samples). We determined similarly up- or downregulated genes in both datasets of liver and gastric cancers, and at least 2 of the three datasets of colon cancer (adjusted $P<0.05$). The derived differential genes were subsequently compared with the consistent genes in PDAC.

RNA Sequencing data

The patients RNA-seq data we used were The Cancer Genome Atlas (TCGA) PDAC data (150 tumors) downloaded from cBioPortal.org and a laser microdissection (LCM) data GSE93326 (n = 65 epithelium vs 65 stroma) downloaded from The National Center for Biotechnology Information Gene Expression Omnibus (NCBI GEO). We retained only genes with an expression value ≥1 in at least half of the samples contained per dataset. To determine differential gene expression, we analyzed TCGA data with *limma* package after log2 transformation, whereas the raw count values for GSE93326 were analyzed with *DESEq2* package (v 1.22.2) in R. Unless otherwise indicated, adjusted $P<0.05$ was used as cut off for differential gene expression. We also included the normal tissue RNA-seq from the Human Protein Atlas (HPA, n = 43 tissues including the pancreas) and the Genotype-Tissue Expression (GTEx, n = 34 tissues including the pancreas). The HPA and GTEx data were both downloaded from the HPA archive version 19 and used as described below.

Determination of normal pancreas expression of the tumor-derived consistent genes

To determine lowly expressed CUGs or high GDGs in normal pancreas, we used the HPA and GTEx data (each contained ≥ 97% of the consistent genes). We extracted the CUGs/CDGs from

each dataset and calculated their median expression values for all other tissues except the pancreas. Genes that their expression in the pancreas was lower than the median for all other tissues were considered lowly expressed in the pancreas, and *vice versa*. Genes that showed low- or high expression in both the HPA and GTEx datasets were selected as consistently low or highly expressed genes in the pancreas. We also analyzed normal tissues in the GSE71729 dataset. Specifically, we compare the combined gene expression of normal liver (*n* = 27), lung (*n* = 19), lymph node (*n* = 10) and spleen (*n* = 11) to that of normal pancreas (*n* = 46, $P<0.01$). Genes with the same expression pattern in GSE71729 as in HPA/GTEx were selected as highly consistent in normal tissues.

PDAC stratification by proliferation, basal-like and classical subtypes

For generating the basal-like and classical subtype subsets, we used the 25 basal-like and 25 classical gene signatures published by Moffitt et al(1). These genes were applied to rank each of TCGA, GSE71729 (Moffitt) and Puleo datasets into the two subsets. For the proliferation high- versus low tumor identification and comparison, we first generated tumor sample data subset containing the expression values of 27 known proliferation genes (**Supplemental Table 5**) from each of TCGA (*n* = 150), GSE71729 (*n* = 145), and Puleo (*n* = 309) datasets. On each subset, we separately applied unsupervised hierarchical clustering and partitioned the tumor samples into proliferation high- and low clusters. Sample numbers were as follows: TCGA – 64 proliferation-high vs 86 low; GSE71729 – 77 proliferation-high vs 68 low; and Puleo – 99 proliferation-high vs 210 low. We then used *limma* package to derive the differentially expressed genes between compared groups. CUGs that emerged as significantly upregulated in at least two of the three datasets ($P<0.05$) were considered high in proliferation-high tumors and *vice versa*. The same rule was applied for CDGs.

Correlation of consistent genes with metastasis

We used three bulk tumor datasets, namely, GSE19279, GSE42952, and GSE71729 to determine the consistent genes correlated with metastasis. With GSE19279 our analysis was liver metastasis samples (*n* = 5) vs liver normal (*n* = 3) or primary PDAC (*n* = 4). With GSE42952, we compared tumor samples of liver metastasis (*n* = 7) vs tumor samples (*n* = 6) from patients that had 'good' prognosis (i.e., longer survival time, *n* = 6) or 'bad' prognosis (i.e., shorter survival time, *n* = 6). On the same dataset, the 'good' or 'bad' prognosis samples were also separately compared to peritoneal metastasis samples (*n* = 4). With GSE71729, the following comparisons were performed: liver metastasis (*n* = 25) vs distant site normal liver tissue samples (*n* = 27); lung metastasis (*n* = 8) versus normal lung samples (*n* = 19) and lymph node metastasis (*n* = 9) vs normal lymph node samples (*n* = 10). In addition, we compared each of the mentioned metastasis tissue samples in GSE71729 to primary PDAC (*n* = 145). Across the respective cohort comparisons, we extracted the CUGs and CDGs that were differentially changed ($P<0.05$).

Differential expression of CUGs and CDGs in mutated versus non-mutated tumor samples

To determine how the frequently mutated genes in PDAC (i.e., *KRAS, TP53, SMAD4* and *CDKN2A*) impact the consistently expressed genes, we extracted the mutation profiles for each tumor sample in TCGA data as obtained from cBioPortal. For *KRAS*, we analyzed for G12D, G12R and G12V since these had considerable sample sizes. Specifically, we performed the

following comparisons: tumors with G12D mutation (*n* = 42), G12V mutation (*n* = 26) and G12R mutation (*n* = 22) each separately compared to no alteration in *KRAS* (*n* = 43). For *TP53*, there were 80 indicated unique mutations of which the most represented (i.e., R175H and R248W) had four samples each. Therefore, we compared all tumors with any *TP53* mutation (*n* = 100) to those with no alteration (*n* = 50). The same approach was applied for *SMAD4* (*n* = 55 mutation vs 95 no alteration) and *CDKN2A* (*n* = 80 mutation vs 70 no alteration). Furthermore, we compared tumors with no alteration in any of these four genes (*n* = 23) to tumors with a mutation in all four (*n* = 24). In each comparison, the identification of the differentially expressed genes was performed as described above for consistent genes, followed by the extraction of the CUGs and CDGs.

Single-cell RNA sequencing analysis
To determine how CUGs and CDGs are expressed in various cell populations, we used our published human scRNA sequencing data of liver metastasis (*n* = 5) (2) or primary PDAC tumor samples (*n* = 61)(2–6). For the metastasis samples, we first derived genes differentially expressed in the tumor epithelial compartment relative to other microenvironmental cell population. These genes were subsequently overlapped with the CUGs and CDGs to extract the common genes.

Pathway and gene ontology analyses
Pathway analyses were performed using DAVID functional annotation platform (v 6.8), or the gene set enrichment analysis tool (GSEA, v 4.0.3) with GSEAPreranked option. Ranking of the genes was based on the product of the logFC and -log(*P*-value) and analysis was based on only genes that were already determined to be significantly altered in the respective comparisons performed. GSEA for Kyoto Encyclopedia of Genes and Genomes (KEGG) pathway, hallmark and transcriptional signatures were run with default parameters, except gene set size filter set at min=10. Gene ontology analyses were performed with DAVID. Details of the number of genes used are indicated in the Figure legends or in Venn diagrams, where applicable.

Assignment of genes to their associated pathways
We assigned the genes to their known functions, e.g., 'metabolism', 'immunity', etc, using published gene lists or gene lists extracted from KEGG or Molecular Signatures Database (MSigDB). For metabolism, we adapted a list of previously published 2,764 metabolic genes (7) and assorted them into metabolic and transporter genes. We also used a published list of 1,988 genes encoding transcriptional regulators in human(8). We compiled a list of signaling genes (n = 2,091 from KEGG), and a list of ~400 immune gene signatures from the Molecular Signatures Database (MSigDB)(9, 10). To the extent possible, these genes were used to assign the consistent genes to their known pathways.

Definition of genes as "high priority" targets
We took two approaches to define the high priority targets, both focusing only on the consistently upregulated genes (CUGs), considering that these are more likely the targets for any therapeutic intervention: a) we searched for genes that are considered potentially essential for PDAC viability based on how interfering with their expression impacted viability in previous CRISPR/Cas9 and RNA interference (RNAi) screens. b) we searched for genes that were not only upregulated but

met other criteria that would suggest they are important targets, namely, low expression in normal pancreas, correlation with high proliferation and poor prognosis including overall survival. First, for priority targets based on effect of gene interference, we analyzed four datasets. Specifically, we used the CRISPR/Cas9 knockout screen data from Project Achilles, GECKO, Behan et al data assessed via BioGRID Open Repository of CRISPR Screens (ORCS)[1.0] each of which covered >17,000 genes. In addition, we used shRNA knockdown screen data from Project Drive (includes ~8,000 genes). For Achilles we analyzed 24 PDAC cell lines with complete dependency scores; for GECKO we analyzed 8 cell lines, for ORCS it was 20 cell lines, and for Project Drive we analyzed 24 cell lines. Coverage of the CUGs was near complete. Specifically, of the 2,010 CUGs, we found 1,962 (~98%) in the Achilles data, 1,947 (~97%) in the GECKO data and 1,916 (95.3%) in Behan et al. ORCS data. Of note, of the genes identified as essential by Project Achilles across over 700 cell lines, 245 overlapped with our CUGs and 137 were CDGs. Also, over 400 CUGs overlapped with the cancer fitness genes defined by Behan et al. The Project Drive, which was originally based on 7,975 genes predicted to be essential, included 972 CUGs (48%). We used the available CUGs per dataset to perform unsupervised hierarchical clustering. We then partitioned each dataset into two clusters: genes that their knockdown strongly impacted viability and genes with no strong impact. Only the CUGs (*n* = 185) that strongly impacted viability in at least three datasets were presented as essential/priority targets for PDAC. In addition to the CRISPR/Cas9 and shRNA screen data, we defined a second set of high priority genes. These include CUGs (*n* = 336) that are “low in normal pancreas” and correlated with high proliferation, and, in addition, correlated with either basal-like subtype or overall survival in at least 1 dataset. Collectively, across the two analyses approach, we identified a total of 450 CUGs that we called “high priority” targets in PDAC.

Cell line stratification and prediction of drug response

The 185 high priority therapeutic targets identified in gene interference screen analysis were used to stratify PDAC cell lines in the cancer cell line encyclopedia (CCLE) (*n* = 44 cell lines)(11) and GSE57083 (AstraZeneca) datasets (*n* = 23 cell lines). In the case of GSE57083, most cell lines existed as biological duplicates and for those cell lines their gene expression data were averaged to generate one datapoint per cell line as in the CCLE data. Thereafter, cell lines in the two datasets were stratified into those that have a high expression of the priority genes and those with low expression using clustering analysis. Following the identification of representative cells in high-expressing or low-expressing subgroups, the therapeutic sensitivity of the high-expressing groups were then determined using the Genomics of Drug Sensitivity in Cancer (GDSC) data(12), which contains experimentally determined sensitivity score for >195 compounds. Compounds that were effective against cell lines expressing a higher level of the high priority genes were selected for further validation studies.

Cell culture, inhibitors and metabolomics

The human PDAC cell lines namely, ASPC1, PA-TU-8902, HPAC and PANC0327 were obtained from the American Type Culture Collection or the German Collection of Microorganisms and Cell Cultures (DSMZ). The mouse PDAC cell line 7940b cells (C57BL/6J strain)(13) was derived from KPC tumor *(Ptf1a-Cre;LSL-Kra^{sG12D}; Trp53$^{flox/+}$*) – also called the KPC cell line. The KPC cell was provided under a material transfer agreement by Dr. Gregory Beatty (University of Pennsylvania,

Philadelphia). The cell lines were mycoplasma-tested (Lonza MycoAlert Plus, LT07-710), used within 10 passages and cultured in DMEM (Gibco, 11965-092) with 10% fetal bovine serum (FBS). The cells were cultured in 37°C incubator under humidified atmosphere. For the inhibitors tested: AZD5153 (Cat# 20864) and gemcitabine (Item# 11690) were obtained from Cayman Chemicals (USA). BRD4 selective degrader BD-9136 was a kind gift from Prof. Shaomeng Wang (University of Michigan). Cell viability experiments were performed using CellTiter-Glo® 2.0 Cell Viability Assay (G9241, Promega) according to manufacturer's instruction. Metabolomics profiling was performed by liquid chromatography tandem mass spectrometry (LC/MS/MS) and analyzed as previously described(14).

Histology and Ki67 staining

Tumor tissue section and Ki-67 staining (antibody Cat# 9027, Cell Signaling Technology) was done according to immunohistochemistry protocol in our lab as previously published(2, 15).

URLs

ArrayExpress database http://www.ebi.ac.uk/arrayexpress
BioGRID ORCS https://orcs.thebiogrid.org/Dataset/114
cBioPortal https://www.cbioportal.org/
DAVID functional annotation platform https://david.ncifcrf.gov/
DepMap https://depmap.org/portal/download/
Functional Annotation Tool https://davidbioinformatics.nih.gov/summary.jsp
Genomics of Drug Sensitivity in Cancer (GDSC) portal https://www.cancerrxgene.org/
Human Protein Atlas https://www.proteinatlas.org/
International Cancer Genome Consortium (ICGC) data portal https://dcc.icgc.org/projects/
KEGG (Kyoto Encyclopedia of Genes and Genomes) https://www.genome.jp/kegg/
NCBI GEO Datasets https://www.ncbi.nlm.nih.gov/geo/
PubMed portal https://pubmed.ncbi.nlm.nih.gov/

References

1. Moffitt RA, et al. Virtual microdissection identifies distinct tumor- and stroma-specific subtypes of pancreatic ductal adenocarcinoma. *Nat Genet*. 2015;47(10):1168–1178.

2. Kemp SB, et al. Pancreatic cancer is marked by complement-high blood monocytes and tumor-associated macrophages. *Life Sci Alliance*. 2021;4(6):e202000935.

3. Steele NG, et al. Multimodal mapping of the tumor and peripheral blood immune landscape in human pancreatic cancer. *Nat Cancer*. 2020;1(11):1097–1112.

4. Lee JJ, et al. Elucidation of Tumor-Stromal Heterogeneity and the Ligand-Receptor Interactome by Single-Cell Transcriptomics in Real-world Pancreatic Cancer Biopsies. *Clin Cancer Res*. 2021;27(21):5912–5921.

5. Werba G, et al. Single-cell RNA sequencing reveals the effects of chemotherapy on human pancreatic adenocarcinoma and its tumor microenvironment. *Nat Commun*. 2023;14(1):797.

6. Peng J, et al. Single-cell RNA-seq highlights intra-tumoral heterogeneity and malignant progression in pancreatic ductal adenocarcinoma. *Cell Res*. 2019;29(9):725–738.

7. Possemato R, et al. Functional genomics reveal that the serine synthesis pathway is essential in breast cancer. *Nature*. 2011;476(7360):346–350.

8. Ravasi T, et al. An Atlas of Combinatorial Transcriptional Regulation in Mouse and Man. *Cell*. 2010;140(5):744–752.

9. Liberzon A, et al. Molecular signatures database (MSigDB) 3.0. *Bioinformatics*. 2011;27(12):1739–1740.

10. Subramanian A, et al. Gene set enrichment analysis: A knowledge-based approach for interpreting genome-wide expression profiles. *Proceedings of the National Academy of Sciences*. 2005;102(43):15545–15550.

11. Barretina J, et al. The Cancer Cell Line Encyclopedia enables predictive modelling of anticancer drug sensitivity. *Nature*. 2012;483(7391):603–607.

12. Yang W, et al. Genomics of Drug Sensitivity in Cancer (GDSC): a resource for therapeutic biomarker discovery in cancer cells. *Nucleic Acids Res*. 2013;41(Database issue):D955-961.

13. Long KB, et al. IFNγ and CCL2 Cooperate to Redirect Tumor-Infiltrating Monocytes to Degrade Fibrosis and Enhance Chemotherapy Efficacy in Pancreatic Carcinoma. *Cancer Discov*. 2016;6(4):400–413.

14. Nwosu ZC, et al. Severe metabolic alterations in liver cancer lead to ERK pathway activation and drug resistance. *EBioMedicine*. 2020;54:102699.

15. Zhang Y, et al. Regulatory T-cell Depletion Alters the Tumor Microenvironment and Accelerates Pancreatic Carcinogenesis. *Cancer Discov*. 2020;10(3):422–439.
